# The 40-year debate: a meta-review on what works for juvenile offenders

**DOI:** 10.1007/s11292-021-09472-z

**Published:** 2021-06-12

**Authors:** Laceé N. Pappas, Amy L. Dent

**Affiliations:** School of Social Ecology, University of California, Irvine, 5300 Social and Behavioral Sciences Gateway, Irvine, CA USA

**Keywords:** Corrections, Juvenile offenders, Meta-analysis, Reentry, Rehabilitation, What works

## Abstract

**Objectives:**

This meta-review integrates the findings of meta-analyses and systematic reviews to explore the effect of intervention programs on recidivism for juvenile offenders.

**Methods:**

The systematic literature search gathered 48 meta-analyses and systematic reviews from 53 research reports, contributing 56 independent effect sizes for analysis. These effect sizes were statistically integrated, and five moderators of theoretical and methodological importance were tested using meta-regression.

**Results:**

On average, intervention programs are associated with a significant reduction in recidivism (*r*_*Φ*_ = −0.09, *p* < 0.001) for juvenile offenders who participate in a program compared to those who do not. However, reductions in recidivism significantly vary between the levels of criminal justice system, characteristics of juvenile offenders, types of program modalities, and methodological quality.

**Conclusions:**

Results indicate that intervention programs can be an effective approach to reducing recidivism for juvenile offenders, especially when combined with a rehabilitative program modality.

**Supplementary Information:**

The online version contains supplementary material available at 10.1007/s11292-021-09472-z.

Criminal justice policy is a topic of enduring importance for scholars, legislators, practitioners, and the public. One particular policy that has been subject to much discussion and debate regards the treatment of juvenile offenders.^1^ Increasing crime rates remain a common perception within criminal justice policy and discourse, despite recent statistics indicating juvenile delinquency is declining (Bouchard & Wong, 2018; Office of Juvenile Justice and Delinquency Prevention [OJJDP], 2020). Yet the effectiveness of the criminal justice system, especially the treatment of justice-involved youth, remains contested. Even with the recent decline in juvenile delinquency, there are still over 43,000 youth housed in correctional facilities across the USA, excluding those serving sentences within the community (Office of Juvenile Justice and Delinquency Prevention [OJJDP], 2019). This population is particularly vulnerable due to contextual factors that often initially place them at risk for delinquency, such as family conflict, homelessness, abuse, and school dropout (Fernandes-Alcantara, 2018). These factors underscore the importance of not only treating youth who come in contact with the justice system but of understanding how and why these treatments work.

The “what works” question has dominated research and theory for over 40 years since Robert Martinson’s (1974) inaugural work on it in correctional programming. This question sparked four decades of intervention research, with meta-analyses and systematic reviews attempting to clarify what works to reduce recidivism for incarcerated individuals as well as to update research as punishment models shifted over time. Such research exploring intervention programs for adult and juvenile offenders revealed only modest effects on recidivism (e.g., Andrews & Bonta, 2006; Andrews et al., 1990; Gendreau & Ross, 1987; MacKenzie, 2006), unexpectedly revealed an increase in recidivism (e.g., Whitehead & Lab, 1989), or have suggested that such programs do work in specific contexts (e.g., Andrews et al., 1990; Andrews & Bonta, 2006; Gendreau et al., 2006).

Two lines of thought continue to guide this debate in research and policy regarding interventions for juvenile offenders.^2^ First, the *rehabilitative model* suggests that intervention programs provide the necessary tools to guide this population toward successful outcomes post-contact with the criminal justice system and provide enhanced public safety (Cullen et al., 2017; Lipsey & Cullen, 2007; Moon et al., 2000). Second, the *punitive model* suggests that by implementing tougher sentences on juvenile offenders (e.g., incarceration or intensive community supervision), they will correct their behavior and be deterred from future criminality by learning from the consequences of their actions (Bouchard & Wong, 2018; Lipsey & Cullen, 2007).

Several existing meta-analyses have tested the effect of intervention programs associated with these different models, yet their results are inconsistent. Some meta-analyses show that there are overall reductions in recidivism for juvenile offenders who participate in intervention programs compared to those who do not (e.g., Bouchard & Wong, 2017; Dowden & Andrews, 2003; Van der Stouwe et al., 2014; Wilson et al., 2017). However, other meta-analyses reveal that programs can have the unintended effect of increasing recidivism for juvenile offenders who participate in a program (e.g., Bouchard & Wong, 2018; Petrosino et al., 2010; Stein et al., 2013). A remaining set of meta-analyses show no effects at all (e.g., Livingstone et al., 2013). Taken together, these discrepancies have produced an unclear picture of what works for juvenile offenders.

Meta-review provides an objective opportunity to clarify this picture by statistically combining the many meta-analyses that have produced discrepant findings on the effect of intervention programs for juvenile offenders. As a result, we conducted a meta-review to comprehensively clarify the longstanding debate about punitive and rehabilitative approaches to programs for this population. In doing so, this meta-review is uniquely positioned to advance discourse among scholars and other stakeholders about the 40-year what works debate.

Before proceeding to moderators of theoretical and methodological importance, we first present distinctions among the three types of reviews of research referred to throughout this paper: systematic review, meta-analysis, and meta-review. These methods for integrating research differ in two main ways. The first difference is in the unit of analysis: In a primary study, the unit of analysis is a participant; in a meta-analysis and systematic review, the unit of analysis is a primary study; in a meta-review, the unit of analysis is a meta-analysis or systematic review. The second difference is in the use of statistics: A meta-analysis statistically combines study outcomes, while a systematic review does not (Lipsey & Wilson, 2001; Uman, 2011). Systematic review is instead defined by its methodological steps (i.e., systematically gathering studies), while meta-analysis is defined by its statistical steps (i.e., statistically integrating and reconciling results of primary studies). A meta-review combines the methodological steps of a systematic review with the statistical integration of results like meta-analysis, while focusing on secondary rather than primary research as the unit of analysis (Barnes et al., 2020; Polanin et al., 2016; Thomson et al., 2013; Vancampfort et al., 2019).^3^

Meta-review not only adopts the statistical approach of meta-analysis but also its goals. When a field of research becomes saturated with conflicting findings and theoretical debates, meta-analysis can statistically summarize and resolve the empirical evidence while revealing factors that impact its magnitude or even direction. A meta-review extends that goal by integrating all relevant meta-analytic evidence to provide stakeholders with the most complete, updated, and accurate information about the effect of interventions along with factors contributing to its variation (Thomson et al., 2013).

With these distinctions and goals in mind, we begin by highlighting the rationale for focusing on moderators in the present meta-review. Its methodology and statistics are then described, followed by its results. We conclude with a discussion of these results along with their theoretical, methodological, and practical importance.

## Rational for moderators

Our overarching goal of this meta-review is to integrate and reconcile findings across the “what works” literature for juvenile offenders. Given discrepant findings in previous reviews of research, a summary effect across them likely obscures the true effect of intervention programs by oversimplifying a multifaceted phenomenon—one that differs in its strength or direction based on many moderators of intervention effectiveness, such as type of offender, criminal justice setting, or program modality. Our meta-review is thus focused on and framed around these moderators to which we briefly turn next. A table of specific hypotheses related to these moderators along with their justification is available in the Supplemental File.

### Type of offender

Several attempts to integrate primary research have explored the impact of interventions on recidivism for specific types of offenders. When this effect has been evaluated statistically, meta-analyses have often focused on one type of offender (e.g., drug or sexual) rather than exploring whether intervention effectiveness differs based on the type of offender (e.g., Dopp et al., 2017; Genovés et al., 2006; Lipsey & Wilson, 1998). The Risk-Needs-Responsivity (RNR) model of rehabilitation has been tested on different types of offenders within meta-analytic literature on programs for juvenile offenders, with evidence to support different effects of them between high-risk and low-risk offenders (e.g., Dowden & Andrews, 1999; Latimer & Dowden, 2003). This meta-review expands the classifications of offenders to account for comparisons across other groups of them, namely, serious or violent, non-serious or non-violent, drug offenders, and sexual offenders.

### Criminal justice setting

The setting in which juvenile offenders receive programs can be classified into two broad categories: institutionalized and noninstitutionalized. Institutionalized settings can refer to incarceration, residential facilities, psychiatric hospitals, or any type of setting where the juvenile is in an out-of-home housing placement under secure supervision. In contrast, noninstitutionalized settings may consist of any community-based setting such as probation, school, and afterschool programs. Some prior meta-analyses have explored the effect of programs for juvenile offenders in the community (e.g., Gottschalk et al., 1987a) or for incarcerated youth (e.g., Garrido & Morales, 2007). However, fewer meta-analyses (e.g., Lipsey & Wilson, 1998) have included moderator analyses to identify the unique impact different criminal justice settings have on the effectiveness of intervention programs to reduce recidivism. Some theories (e.g., social learning theory) would suggest that treating juvenile offenders in an institutionalized setting may increase their likelihood of committing crime due to the learned criminality that often perpetuates a criminogenic effect among incarcerated people. Yet other theories (e.g., RNR, deterrence theory) suggest that institutionalized settings might foster better outcomes for juvenile offenders due to the amount of targeted programming and treatment that institutionalized offenders experience over non-institutionalized offenders. The potentially differing impact of these two criminal justice system settings may be an additional factor to consider when deciding a course of treatment for a justice-involved youth.

### Criminal justice system exposure

The criminal justice system operates much like a funnel with programs for juvenile offenders implemented anywhere along the spectrum of being exposed to the criminal justice system. For example, programs can be implemented to divert youth from the system, during their time serving a sentence in a correctional facility or in the community, and during the process of reentering into the community. As such, “criminal justice system exposure” reflects where in this funnel a program is implemented. Research on programs for juvenile offenders focuses on diversion programs and programs that take place inside of correctional facilities for incarcerated juveniles. Only recently have reentry and aftercare programs for justice-involved youth entered the meta-analytic landscape. While meta-analyses have explored each of these domains separately, statistically comparing different forms of justice system exposure has been examined less. Thus, it is an important time to compare across these forms of exposure given the possibility that they shape juvenile experiences and outcomes in the justice system differently. For example, diverting youth from formal processing is a recently popular alternative to incarceration, especially for low-level offenders (e.g., Bouchard & Wong, 2017) because of the negative implications often associated with serving a correctional sentence and then reentering society. Depending on how much exposure a youth has to the justice system, however, there may be important differences in the type or amount of support provided to them that could then shape recidivism. In this vein, exploring how different forms of exposure to the justice system relates to a reduction in recidivism can aid researchers and practitioners in identifying the support provided at each level of justice system exposure and how to better maintain that support for these youth.

### Type of program modality

Several program modalities have been implemented in interventions for juvenile offenders over the last four decades, namely family-based treatment, multi-systemic therapy (MST), cognitive behavioral therapy (CBT), educational, restorative justice (RJ), specialized courts, diversion, intensive-supervision probation, wilderness treatment, and shock incarceration. Some reviews have examined the effect on recidivism across program modalities (e.g., Lipsey, 2009), while others have focused solely on the effect of an individual modality (e.g., specialized courts, Stein et al., 2013; MST, Van der Stouwe et al., 2014). Some programs, such as CBT, are more heavily supported in practice given their proliferation as part of the RNR best practices approach and a larger body of research that exists on them supporting their effectiveness (e.g., Landenberger & Lipsey, 2005). As such, a moderator analysis on program modality is necessary to understand the nuanced effect of each modality across all the literature as well as to compare the relative magnitude of the effect on recidivism among these modalities.

### Methodological quality

Meta-analyses can impact the development of policy and practitioner decisions (Polanin et al., 2016), perhaps especially with regard to effective programs for juvenile offenders. However, critics highlight the low methodological quality of some meta-analyses (Shea et al., 2009) as a separate concern from the methodological quality of the primary research they integrate. As a consequence, policy and practitioner decisions may be erroneously made about the effect of a program based on compromised meta-analytic findings. This possibility reflects the importance of assessing methodological quality of meta-analyses, which has yet to be explored in this area until now.

It is unlikely that all meta-analyses and systematic reviews on intervention programs for juvenile offenders will be of high methodological quality. For example, some of the included systematic reviews and meta-analyses were conducted before best practices were established. It is also unlikely that different levels of methodological quality will produce the same association with recidivism for intervention programs. Some factors that might impact and inflate effect sizes for lower quality reviews are publication bias and selection bias. For example, a best practice is to have multiple researchers screening studies for inclusion and to have multiple coders independently extracting data from included studies (Higgins & Green, 2011). When only one researcher is involved at either of these steps, systematic selection bias may produce an artificial difference in effect sizes among low-, moderate-, and high-quality systematic reviews or meta-analyses. Reviews of lower quality likely take fewer steps to lessen bias and, as a result, may possibly produce inflated effects of interventions on recidivism.

## The present research

Inspired by a longstanding urgency to improve the lives of juvenile offenders, the present meta-review integrates, reconciles, and clarifies four decades of research on the effect of intervention programs for this vulnerable population. By statistically integrating and reconciling the results of previous meta-analyses and systematic reviews, this meta-review provides unique insight into the impact of different programs on recidivism among juvenile offenders. Through five novel moderator analyses, we explore how the effect of these programs varies as a function of important contextual factors such as offender type, criminal justice system setting, criminal justice system exposure, program modality, and methodological quality.

This meta-review also complements previous reviews of research in four main ways. First and foremost, this meta-review offers a statistical integration of effect sizes and moderator analyses to explore variation among them that have both been absent in previous attempts to integrate reviews of research. Second, this meta-review broadens the range of intervention programs to include diversion and aftercare/reentry programs, thereby allowing for a more comprehensive analysis of the available interventions for juvenile offenders. Third, this meta-review focuses on juvenile offenders and does not include meta-analyses or systematic reviews that also included adults, thereby enabling a clearer picture of the impact of these programs specifically for justice-involved youth. Fourth, this meta-review reflects the most current research by also accounting for updates to meta-analyses included in other reviews of research. Taken together, this meta-review builds upon previous attempts to integrate reviews of research and charts a new course for that methodological process in our field.

## Method

Our meta-review adopts similar strategies seen in criminology (e.g., Barnes et al., 2020), psychology (e.g., Bakker et al., 2012; Nuijten et al., 2018), psychiatry (e.g., Vancampfort et al., 2019), and neuroscience (e.g., Button et al., 2013). Rigorously integrating results from meta-analyses is the most appropriate approach to achieve our goals for both methodological and statistical reasons. Methodologically, for example, many of the meta-analyses included in this meta-review are topic specific (i.e., focusing on one type of intervention or one type of offender) and thus better enable identification of moderators waiting to be uncovered in such a large and complex empirical literature (Barnes et al., 2020). Statistically, for example, estimated effects from meta-analyses are often more stable than those from primary studies (Barnes et al., 2020; Lipsey & Wilson, 2001). In the following sections, we detail the methodological and statistical steps of this meta-review.

### Strategy for searching the literature

Like a traditional systematic review, this meta-review adopted multiple complementary strategies to gather the relevant literature for extraction and analysis of data: (1) searching electronic databases, (2) contacting researchers in the field, and (3) hand searching the reference lists of relevant texts (Higgins & Green, 2011). The first strategy was comprehensively searching electronic databases (between July and September of 2018) with terms generated through an iterative and innovative process available in the Supplemental File. The second strategy was included to reduce the potential for publication bias, which involved contacting researchers in the field. We individually contacted thirty scholars who authored at least two meta-analyses or systematic reviews on programs for juvenile offenders to gather reviews that never made it to publication and/or were potentially in the publication pipeline. The third strategy involved hand searching the reference lists of relevant texts as well as hand searching the American Society of Criminology website for conference papers. Taken together, these three search strategies generated 2232 documents in total to be retrieved for the screening process, which is discussed next.

### Inclusion and exclusion criteria

Studies for this meta-review were included based on certain criteria. After pilot testing the list of search terms, we created an abstract screening tool in order to screen for information that would be clearly and consistently reported in the abstracts. Using the PICOS framework (Uman, 2011) that guides the inclusion and exclusion criteria for this meta-review,^4^ the abstract screening tool was designed with questions that would make disqualifying studies easiest to determine as the first questions. We retained meta-analyses and systematic reviews for further review of the full text if they were a review of research, not a primary study, a review on juvenile or youth offenders, the abstract was written in English, and the efficacy and/or effectiveness of a program was tested. A complete list of the abstract screening questions is provided in the Supplemental File. The lead researcher screened all abstracts to determine which studies would be considered further for inclusion.

After removing duplicates, we then examined the 214 reviews of research that met the abstract screening criteria in their entirety. Specifically, the “Method” and “Results” sections were evaluated based on a full-text screening guide we iteratively developed and pilot tested, which contained more detailed questions about whether they would qualify for the meta-review. The final version of this tool is provided in the Supplemental File. As with the screening of abstracts, the lead researcher screened the full texts, and if any concerns arose over including a meta-analysis or systematic review, we consulted one independent substantive expert and one independent methodological expert.

After we pilot tested the full-text screening guide, we then proceeded to screen the full text of reports. Each meta-analysis or systematic review must have answered “yes” to the following six inclusion criteria:

1. Met the qualifying criteria for a systematic review on item 4 of AMSTAR-2 (answered at least “partial yes” in addition to meeting the substantive criteria; Shea et al., 2017).
2. Focused exclusively on juvenile offenders between the ages of 10 and 25.
3. Examined any program as long as the juvenile offender had made contact with the criminal justice system.
4. Examined interventions that had a treatment condition where the juvenile offender received a type of program and a comparison condition where the juvenile offender did not experience that type of program.^5^
5. Measured recidivism broadly, including new contact with law enforcement, rearrests, reconviction, and/or probation violations.^6^
6. Reported in English.

Justification for these criteria can be found in the Supplemental File.

Three exclusion criteria accompanied these six inclusion criteria. First, meta-analyses and systematic reviews were excluded if the participants were aged 26 and over or they included adults in addition to juveniles (e.g., Landenberger & Lipsey, 2005; Latimer et al., 2005; Mitchell et al., 2012).^7^ Second, reviews were excluded if there was not enough information to calculate an effect size with authors contacted when necessary to retrieve this unreported information. Third, meta-analyses and systematic reviews that report their abstracts in English but their full text in another language were also excluded from this meta-review. The Supplemental File contains a complete list of excluded studies.

**Fig. 1:**
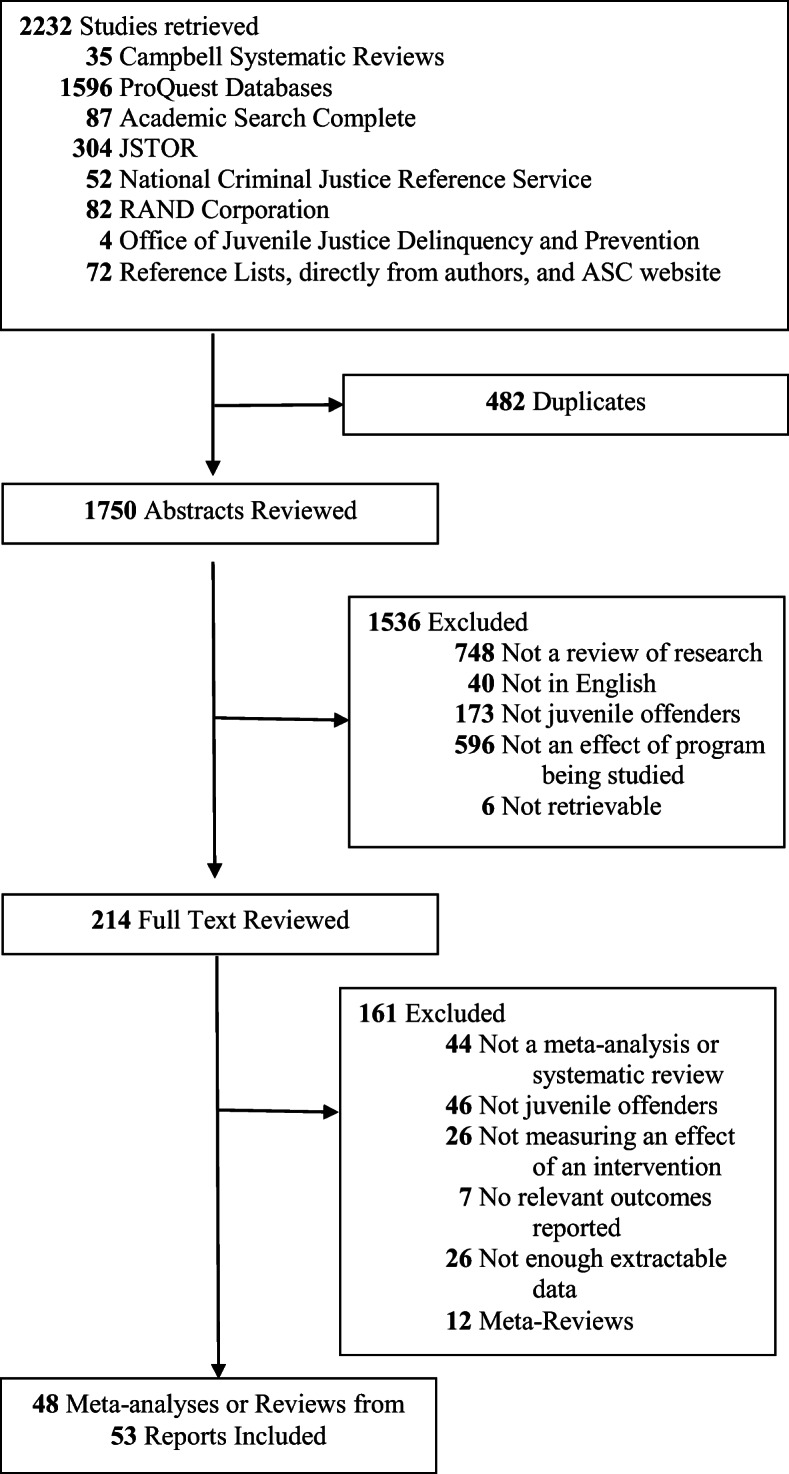
Flow diagram of study selection

Ultimately, 53 meta-analyses and systematic reviews (see Fig. 1) qualified for this meta-review after the abstract and full text screening phases were completed based on these inclusion and exclusion criteria.

### Procedure for gathering information about studies

We adapted a coding protocol to extract all relevant information for the overall and moderator analyses, which included report characteristics, setting characteristics, participant (review of research study) characteristics, program characteristics, and information about the outcome variables. The coding protocol, along with the process for its pilot testing and the rational for its response options, is provided in the Supplemental File.

Coding procedure. The lead researcher and a trained research assistant independently coded all included meta-analyses and systematic reviews. Research assistants received 15 h of training and practice coding. Any discrepancies in the extraction of the data that arose between the lead researcher and the research assistant were reconciled through discussion (Borenstein et al., 2009; Lee & Wong, 2020). The research team contacted authors to gather any relevant missing data and to reconcile any discrepancies in their data.

### Assessment of methodological quality

In traditional meta-analyses and systematic reviews, assessing methodological quality of the included primary studies often relates to their type of design (e.g., pre/post, quasi-experimental, randomized control trial) using scales such as the Maryland Scale (see Bouchard & Wong, 2017; Lee & Wong, 2020). When assessing the methodological quality of a meta-analysis or systematic review, there are additional factors to consider, such as description and justification for the PICOS framework, description of a priori methods, information on multiple screeners/coders, risk of bias assessments, publication bias assessments, heterogeneity assessments, conflicts of interest, appropriate methods for statistical synthesis, and description of included and excluded studies. Using the updated AMSTAR-2 tool (Shea et al., 2017), each meta-analysis and systematic review included in this meta-review was independently scored on all AMSTAR-2 items by the lead researcher and the research assistant coding that review. Any discrepancies in the appraisal of methodological quality were also resolved through discussion. Each review of research was assigned a grade of low, moderate, or high based on its AMSTAR-2 scoring.

### Method of data integration

Effect size estimation. Many of the meta-analyses examined during the preliminary phase of this project used different effect sizes. The three effect sizes most often reported were a Cohen’s *d*-index, an odds ratio, and a phi coefficient. A phi coefficient is a Pearson’s product-moment correlation coefficient for dichotomous measures, such as *treatment* versus *control* and *recidivated* versus *did not recidivate* (e.g., Andrews & Dowden, 2006; Lipsey, 2009; Lipsey & Wilson, 2001). For this meta-review, meta-analytic studies that include *d-*indices and odds ratios were converted to phi coefficients. Converting them to a phi coefficient, which appears most frequently in the literature, best standardizes different effect sizes on recidivism into a common statistical language (Lipsey & Cullen, 2007). While the phi coefficient can present smaller numerical values, they can still have practical significance. Phi coefficients can easily be translated into a percent change in recidivism, which is reported alongside statistical results for comparative relevance to the field and to not minimize a meaningful effect size from what might look like a modest value (Lipsey, 2009; Lipsey & Cullen, 2007).^8^ The effect sizes are interpreted according to conventional and empirical benchmarks relative to other findings in the field, with negative values reflecting a correlation between juvenile offenders participating in a program and a reduction in recidivism (Kim et al., 2013; Lipsey & Cullen, 2007).

Statistical approach. We conducted all analyses using the Comprehensive Meta-Analysis (CMA) software, version 3.0. We adopted two approaches to conducting moderator analyses. First, we tested moderators using subgroup analysis without adjusting for covariates. Second, we tested moderators using meta-regression techniques, adjusting for two covariates in the reviews: (1) methodological quality and (2) publication status. While the results of both models are reported, we interpret the meta-regression results because the inclusion of these covariates strengthens the trustworthiness of results (that they are not an artifact of methodological quality and publication bias).

Calculating mean effect sizes. A weighting procedure was applied to calculate all phi coefficients for the overall and moderator analyses (Borenstein et al., 2009). Larger sample sizes contribute more precise estimates of population parameters and should be given more weight to account for this precision. A 95% confidence interval was constructed around each mean phi coefficient in both the overall and moderator analyses.

Identifying independent samples. More than one effect size can be found in a report, especially when using meta-analyses as the unit of analysis. Having more than one effect size in a given report raises the question of what qualifies as an independent sample because each independent sample can only contribute one effect size to the summary effect or moderator category mean. If there are multiple effect sizes from the same sample, they are not independent of each other. One solution is to calculate an average effect size from the multiple effect sizes found in each sample, so that each sample only contributes one effect size to the summary effect or moderator category mean (Lipsey & Wilson, 2001). We adopted this shifting unit of analysis (Cooper, 2010) approach to (1) minimize violations to the independent samples assumption while still retaining as much data as possible and (2) minimize bias from estimates calculated from small samples. To calculate these average effect sizes, we coded each phi coefficient reflecting the relation between participating in a program and recidivism as a separate estimate, but then averaged all phi coefficients from a single sample prior to finding the summary effect. For moderator analyses, we did not average together phi coefficients from the same sample that belonged to different categories of the moderator. For example, if different effect sizes between a program and recidivism could be calculated for institutionalized and non-institutionalized offenders from a single review, they would contribute one (averaged) phi coefficient to the summary result. For the moderator analysis on criminal justice setting, however, this review would contribute one phi coefficient to the institutionalized category and one to the noninstitutionalized category.^9^ Relatedly, if the results of a meta-analysis or systematic review were presented in two different reports, they were treated as a single review. The most complete report was coded as the main review, where other overlapping reports provided additional information for data extraction and are indicated in the reference list as an included review (though not an independent sample). In reviews that included multiple meta-analyses, distinguishably different samples were identified and coded as independent (e.g., see Bouchard & Wong, 2018; Wilson et al., 2003).

Testing for moderators. Heterogeneity analyses were conducted on all moderator analyses to determine whether the association (phi coefficient) between programs for juvenile offenders and recidivism differs based on factors of theoretical, practical, or methodological importance. As discussed previously, some of those factors include the type of juvenile offender, criminal justice system setting, criminal justice system exposure, type of program modality, and methodological quality of the included reviews. Heterogeneity analysis compares the observed variation in phi coefficients with the amount of variation that would be explained through sampling or estimation error alone (Borenstein et al., 2009). If effect sizes varied significantly more than would be expected under these assumptions, moderator analyses were conducted to determine how the strength and/or direction of phi coefficients differed due to our proposed moderators. A within-group goodness-of-fit statistic (*Q*_*w*_) was used to test total variation around the summary effect of the phi coefficients in this meta-review. A significant *Q*_*w*_ value implies there is more variation around the summary effect than chance or error alone can explain, offering empirical justification that the moderator analysis should be conducted (Borenstein et al., 2009; Lipsey & Wilson, 2001).

We used a between-groups goodness-of-fit statistic (*Q*_*b*_) when conducting the meta-regression moderator analyses. A significant *Q*_*b*_ value implies there is more variation among mean phi coefficients for the categories of the moderator than can be explained by chance alone. For example, a significant *Q*_*b*_ value in a moderator analysis based on the type of juvenile offender signals that the mean phi coefficient differs depending on whether the youth committed a sexual, serious, nonserious, or drug offense. Pairwise comparisons were then conducted to test differences between the categories of each moderator.

Modeling error. Both the overall and moderator analyses initially adopted two different models of error. A fixed effect model assumes that the phenomena have only one true effect in the population, and any variation we see in studies is the result of sampling or measurement error. In contrast, a random effects model assumes that there are multiple true effects in the population and variation we see in studies reflects (in part) real differences in the population (Borenstein et al., 2009). Strong theoretical and empirical evidence suggests that there is not one true effect of interventions for juvenile offenders, but rather many based on moderators—the assumption underlying random-effects modeling in meta-analysis (Borenstein et al., 2009). The exploration of moderators to address this complexity and the potential for more than one source of true variation can only be accomplished through a random-effects model. As such, only random-effects models are reported and interpreted, with the results of the fixed-effects models provided in the Supplemental File.

## Results

### Intervention programs

The literature search resulted in 48 meta-analyses and systematic reviews from 53 research reports, including 3105 primary studies, with at least one phi coefficient reflecting the relation between juvenile offenders participating in an intervention program and recidivism. These 53 reviews produced a total of 80 relevant phi coefficients with 56 samples considered independent for analysis. We transformed all phi coefficients used for analyses into *z* scores by applying a Fisher’s transformation.^10^ A summary of the data coded from these reviews for the overall analysis can be found in the Supplemental File.

Publication bias. We conducted meta-regression moderator analyses on two types of publication bias: publication type and publication status. For the first type of publication bias, we categorized effect sizes into five groups to reflect the publication type of the included reviews: private reports, government reports, Campbell/Cochrane Collaboration systematic reviews, book chapters, and journal articles. The association between juvenile offenders participating in an intervention program and recidivism did not vary significantly based on publication type (*Q*_*b*_(4) = 1.06, *p* = 0.901). For the second type of publication bias, we categorized effect sizes into two groups to reflect the publication status of the included reviews: peer-reviewed and non-peer-reviewed. The association between participating in a program and recidivism also did not vary significantly based on publication status (*Q*_*b*_(1) = 0.49, *p* = 0.483). Although there were no statistically significant differences, average phi coefficients were stronger for reviews that were not peer reviewed (*r*_*Φ*_ = −0.113, 95%CI (−0.186, −0.039), *p* = 0.003, *k* = 5) than for those that were peer-reviewed (*r*_*Φ*_ = −0.085, 95%CI (−0.107, −0.063), *p* < 0.001, *k* = 51).

Overall phi coefficient with recidivism. We used summary phi coefficients from each meta-analysis or systematic review to calculate the overall relation between juvenile offenders participating in an intervention program and recidivism in this meta-review. We first averaged together reviews that included multiple summary effect sizes with overlapping samples to create one independent effect size that would contribute to the overall phi coefficient for this meta-review. A total of 56 independent phi coefficients contributed to the summary relation between juvenile offenders participating in an intervention program and recidivism in this meta-review. Overall, we found a significant mean weighted phi coefficient under the random effects model (*r*_*Φ*_ = −0.087, 95%CI (−0.108, −0.065), *p* < 0.001, *k* = 56). Translating this summary effect into a more meaningful marker of impact (Lipsey & Cullen, 2007), there is a (average) 17.4% difference in recidivism rates between juvenile offenders who do and do not participate in a program. However, there was significant variation around this summary effect (*Q*_*w*_(55) = 4371.090, *p* < 0.001).

Meta-regression moderator analyses. This significant *Q*_*w*_ statistic indicates that there is more variation around the 56 average phi coefficients (one for each independent sample from each systematic review or meta-analysis) than random error would suggest, supporting the decision to conduct moderator analyses (in addition to our substantive reasons for doing so). We thus conducted five moderator analyses to explore how variables of theoretical, practical, and methodological importance change the association between participating in an intervention program and recidivism. Table 1 presents the complete results from these moderator analyses, while results described below are from the random effects meta-regression models unless otherwise reported.

**Table 1:** Results from the random effects overall and moderator analyses

	Adjusted effects^a^				Unadjusted effects^b^			
		*Q*_*b*_				*Q*_*b*_		
	*r*_*Φ*_	95%CI	% Change^d^	*k*^c^	*r*_*Φ*_	95%CI	% Change	*k*
Overall phi	−0.087	(−0.108, −0.065)	−17.40%	56	−0.087	(−0.108, −0.065)	−17.40%	56
Moderators								
Offender type		26.69***				18.808**		
Drug	−0.026	(−0.091, 0.039)	−5.24%	4	−0.025	(−0.046, −0.004)	−5.03%	4
General	−0.045	(−0.080, −0.009)	−8.90%	32	−0.071	(−0.097, −0.045)	−14.14%	32
Non-serious/non-violent	−0.061	(−0.109, −0.013)	−12.26%	8	−0.066	(−0.086, −0.046)	−13.18%	8
Serious/violent	−0.127	(−0.175, −0.080)	−25.48%	8	−0.140	(−0.201, −0.078)	−28.07%	8
Sexual	−0.153	(−0.220, −0.085)	−30.54%	4	−0.174	(−0.362, 0.027)	−34.88%	4
CJ system setting		19.55**				12.459***		
Institutionalized	−0.127	(−0.170, −0.083)	−25.32%	33	−0.132	(−0.168, −0.096)	−26.49%	33
Noninstitutionalized	−0.045	(−0.078, −0.012)	−9.02%	30	−0.052	(−0.078, −0.025)	−10.33%	30
CJ system exposure		20.74**				17.661***		
Correctional	−0.127	(−0.175, −0.079)	−25.40%	14	−0.125	(−0.155, −0.096)	−25.10%	14
Diversion	−0.036	(−0.075, 0.002)	−7.28%	19	−0.049	(−0.082, −0.016)	−9.79%	19
Reentry/Aftercare	−0.035	(−0.094, 0.024)	−7.02%	8	−0.040	(−0.073, −0.007)	−7.94%	8
Program modality		59.54***				32.201***		
Cognitive behavioral treatment	−0.099	(−0.202, −0.007)	−19.82%	10	−0.102	(−0.142, −0.061)	−20.32%	10
Diversion treatment	−0.056	(−0.116, −0.032)	−11.20%	4	−0.062	(−0.087, −0.038)	−12.49%	4
Educational	−0.055	(−0.137, 0.028)	−11.00%	3	−0.041	(−0.079, −0.004)	−8.27%	3
Family-based treatment	−0.115	(−0.165, −0.064)	−22.90%	5	−0.137	(−0.210, −0.063)	−27.49%	5
Intensive supervision probation	0.003	(−0.095, 0.101)	0.62%	2	0.004	(−0.055, 0.062)	0.74%	2
Multisystemic treatment	−0.231	(−0.299, −0.164)	−46.28%	4	−0.152	(−0.358, 0.069)	−30.32%	4
Restorative justice	−0.074	(−0.116, −0.032)	−14.70%	6	−0.083	(−0.139, −0.027)	−16.69%	6
Shock incarceration	0.041	(−0.024, 0.107)	8.24%	2	0.022	(−0.044, 0.087)	4.34%	2
Specialized courts	−0.043	(−0.086, 0.000)	−8.62%	6	−0.027	(−0.046, −0.009)	−5.44%	6
Wilderness therapy	−0.105	(−0.202, −0.007)	−20.90%	1	−0.090	(−0.131, −0.048)	−18.00%	1
Methodological quality		5.74				4.355		
Low	−0.126	(−0.169, −0.083)	−25.18%	11	−0.129	(−0.184, −0.074)	−25.88%	11
Moderate	−0.084	(−0.115, −0.052)	−16.70%	21	−0.070	(−0.080, −0.059)	−13.92%	21
High	−0.068	(−0.097, −0.038)	−13.50%	24	−0.068	(−0.094, −0.042)	−13.66%	24

^a^Adjusted effects = meta-regression results controlling for publication bias and methodological quality

^b^Unadjusted effects = traditional moderator analysis without methodological covariates

^c^*k* = number of samples considered independent for analysis

^d^Difference between the recidivism rate for the intervention and a control recidivism rate (see Lipsey & Cullen, 2007)

**p < 0.01, ***p < 0.001

The following meta-regression results compare juvenile offenders who participate in an intervention program to those who do not, while controlling for publication status (peer-reviewed versus non peer-reviewed) and methodological quality (AMSTAR-2 grade) of the included systematic reviews and meta-analyses. When conducting each moderator analysis, an average phi coefficient was calculated for each category of that moderator (e.g., offender type). These average phi coefficients reflect the strength and direction of association between participating (yes/no) in an intervention program and recidivism (yes/no). A positive phi coefficient indicates that program participation is associated with increased recidivism, while a negative phi coefficient indicates that program participation is associated with a reduction in recidivism. The regression coefficients represent pairwise comparisons, which reflect a difference in strength of the recidivism reduction between two categories of the moderator, such as two different types of offenders (see Table 2). We turn to the results of that moderator analysis next.

**Table 2:** Random effects pairwise comparison matrix for type of offender moderator

	Reference group				
Comparison group	General	Sexual	Serious	Nonserious	Drug
General	--	0.001	0.001	0.542	0.602
Sexual	−0.108	--	0.514	0.021	0.005
Serious	−0.083	0.025	--	0.038	0.009
Nonserious	−0.017	0.091	0.067	--	0.370
Drug	0.018	0.127	0.101	0.035	--

Numbers below the diagonal reflect the regression coefficient for each pairwise comparison; numbers above the diagonal reflect the *p*-value for each pairwise comparison

### Moderators

#### Offender type

We categorized effect sizes into five types of juvenile offenders who participated in an intervention program: sexual, serious or violent, non-serious or non-violent, general, and drug. The association between participating in a program and recidivism varied significantly based on the type of offender (*Q*_*b*_(7) = 26.69, *p* < 0.001). Compared to juvenile offenders who did not participate in a program, participating in an intervention program is most strongly associated with a reduction in recidivism for sexual offenders (*r*_*Φ*_ = −0.153, 95%CI (−0.220, −0.085), *p* < 0.001, *k* = 4) and serious or violent offenders (*r*_*Φ*_ = −0.127, 95%CI (−0.175, −0.080), *p* < 0.001, *k* = 8). Participating in an intervention program is less strongly associated with a reduction in recidivism for non-serious or non-violent offenders (*r*_*Φ*_ = −0.061, 95%CI (−0.109, −0.013), *p* = 0.013, *k* = 8) and general offenders (*r*_*Φ*_ = −0.045, 95%CI (−0.080, −0.009), *p* = 0.013, *k* = 32). The weakest associations with reduced recidivism are for drug offenders (*r*_*Φ*_ = −0.026, 95%CI (−0.091, 0.039), *p* = 0.432, *k* = 4).

Six of the pairwise comparisons revealed significant differences in recidivism between the two groups being tested (see Table 2 for all pairwise comparisons). Most notably, both sexual offenders and serious offenders had a significantly stronger reduction in recidivism than general, nonserious, and drug offenders.

#### Criminal justice setting

We categorized effect sizes into two types of criminal justice settings: institutionalized and non-institutionalized. The association between participating in an intervention program and recidivism varied significantly based on the type of setting (*Q*_*b*_(5) = 19.55, *p* = 0.002). Compared to juvenile offenders who do not participate in a program, participating in an intervention program is more strongly associated with a reduction in recidivism for offenders in an institutionalized setting (*r*_*Φ*_ = −0.127, 95%CI (−0.170, −0.083), *p* < 0.001, *k* = 33) than for offenders in a non-institutionalized setting (*r*_*Φ*_ = −0.045, 95%CI (−0.078, −0.012), *p* = 0.007, *k* = 30). A pairwise comparison revealed a statistically significant difference in recidivism between these two settings (*b* = 0.082, *p* < 0.001).

#### Criminal justice system exposure

We categorized effect sizes into three levels of criminal justice system exposure: diversion, correctional, and reentry/aftercare. The association between participating in an intervention program and recidivism varied significantly based on the level of criminal justice system exposure (*Q*_*b*_(6) = 20.74, *p* = 0.002]. Compared to juvenile offenders who do not participate in a program, participating in an intervention program is most strongly associated with a reduction in recidivism for correctional programs (*r*_*Φ*_ = −0.127, 95%CI (−0.175, −0.079), *p* < 0.001, *k* = 14). Participating in a program is less strongly associated with a reduction in recidivism for diversion programs (*r*_*Φ*_ = −0.036, 95%CI (−0.075, 0.002), *p* = 0.062, *k* = 19) and reentry/aftercare (*r*_*Φ*_ = −0.035, 95%CI (−0.094, 0.024), *p* = 0.243, *k* = 8).

Pairwise comparisons (see Table 3) revealed statistically significant differences between correctional and diversion programs (*b* = −0.091, *p* = 0.001) as well as between correctional and reentry/aftercare programs (*b* = −0.092, *p* = 0.009).

**Table 3:** Random effects pairwise comparison matrix for criminal justice system exposure moderator

	Reference group		
Comparison group	Diversion	Correctional	Reentry/Aftercare
Diversion	--	0.000	0.970
Correctional	−0.091	--	0.009
Reentry/Aftercare	0.001	0.092	--

Numbers below the diagonal reflect the regression coefficient for each pairwise comparison; numbers above the diagonal reflect the *p*-value for each pairwise comparison

#### Program modality

We categorized effect sizes into ten types of program modality used to treat a juvenile offender: multisystemic therapy (MST), family-based treatment, wilderness therapy, cognitive behavioral therapy (CBT), restorative justice (RJ), diversion, educational, specialized courts, intensive supervision probation (ISP), and shock incarceration.^11^ The association between participating in an intervention program and recidivism varied significantly based on the program modality (*Q*_*b*_(13) = 59.54, *p* < 0.001). Compared to juvenile offenders who do not participate in a program, participating in an intervention program is most strongly associated with a reduction in recidivism for MST programs (*r*_*Φ*_ = −0.231, 95%CI (−0.299, −0.164), *p* < 0.001, *k* = 4) and family treatment programs (*r*_*Φ*_ = −0.115, 95%CI (−0.165, −0.064), *p* < 0.001, *k* = 5). Participating in an intervention program is less strongly associated with a reduction in recidivism for wilderness therapy (*r*_*Φ*_ = −0.105, 95%CI (−0.202, −0.007), *p* = 0.036, *k* = 1) and CBT programs (*r*_*Φ*_ = −0.099, 95%CI (−0.202, −0.007), *p* < 0.001, *k* = 10). Average effect sizes are relatively weaker for RJ (*r*_*Φ*_ = −0.074, 95%CI (−0.116, −0.032), *p* = 0.001, *k* = 6), diversion programs (*r*_*Φ*_ = −0.056, 95%CI (−0.116, −0.032), *p* < 0.001, *k* = 4), educational programs (*r*_*Φ*_ = −0.055, 95%CI (−0.137, 0.028), *p* = 0.187, *k* = 3), and specialized courts (*r*_*Φ*_ = −0.043, 95%CI (−0.086, 0.000), *p* = 0.051, *k* = 6). Conversely, participating in a program is associated with increased recidivism for ISP (*r*_*Φ*_ = 0.003, 95%CI (−0.095, 0.101), *p* = 0.950, *k* = 2) and shock incarceration (*r*_*Φ*_ = 0.041, 95%CI (−0.024, 0.107), *p* = 0.217, *k* = 2).

Seventeen of the pairwise comparisons revealed significant differences in recidivism between the two groups being tested (see Table 4 for all pairwise comparisons). Most notably, multisystemic therapy programs had a significantly stronger reduction in recidivism than for every other type of program modality.

**Table 4:** Random effects pairwise comparison matrix for program modality moderator

	Reference group									
Comparison group	CBT	Diversion	Educational	Family	ISP	MST	RJ	Shock	SC	Wilderness
CBT	--	0.132	0.338	0.604	0.057	0.000	0.352	0.000	0.045	0.916
Diversion	0.043	--	0.983	0.063	0.281	0.000	0.561	0.012	0.667	0.362
Educational	0.044	0.001	--	0.221	0.371	0.001	0.696	0.070	0.796	0.443
Family	−0.016	−0.058	−0.059	--	0.036	0.002	0.181	0.000	0.026	0.851
ISP	0.102	0.059	0.058	0.118	--	0.000	0.159	0.527	0.398	0.127
MST	−0.132	−0.175	−0.176	−0.117	−0.235	--	0.000	0.000	0.000	0.027
Restorative justice (RJ)	0.026	−0.017	−0.018	0.041	−0.077	0.158	--	0.002	0.312	0.560
Shock incarceration	0.140	0.097	0.096	0.156	0.038	0.273	0.115	--	0.032	0.013
Specialized courts (SC)	0.056	0.013	0.012	0.072	−0.046	0.188	0.030	−0.084	--	0.240
Wilderness	−0.005	−0.048	−0.049	0.010	−0.108	0.127	−0.031	−0.146	−0.061	--

Numbers below the diagonal reflect the regression coefficient for each pairwise comparison; numbers above the diagonal reflect the *p*-value for each pairwise comparison

#### Methodological quality

We categorized effect sizes into three levels of methodological quality for the meta-analyses and systematic reviews included in this meta-review: low, moderate, and high. The association between participating in an intervention program and recidivism did not vary significantly based on methodological quality (*Q*_*b*_(3) = 5.74, *p* = 0.125). Compared to juvenile offenders who do not participate in a program, participating in an intervention program is most strongly associated with a reduction in recidivism for low-quality reviews (*r*_*Φ*_ = −0.126, 95%CI (−0.169, −0.083), *p* < 0.001, *k* = 11). Participating in a program is less strongly associated with a reduction in recidivism for moderate-quality reviews (*r*_*Φ*_ = −0.084, 95%CI (−0.115, −0.052), *p* < 0.001, *k* = 21). The relatively weakest average association is for high-quality reviews (*r*_*Φ*_ = −0.068, 95%CI (−0.097, −0.038), *p* < 0.001, *k* = 24). Pairwise comparisons (see Table 5) only revealed statistically significant differences between low-quality reviews and high-quality reviews (*b* = 0.058, *p* = 0.025).

**Table 5:** Random effects pairwise comparison matrix for AMSTAR moderator

	Reference group		
Comparison group	Low	Moderate	High
Low	--	0.110	0.025
Moderate	0.042	--	0.463
High	0.058	0.016	--

Numbers below the diagonal reflect the regression coefficient for each pairwise comparison; numbers above the diagonal reflect the *p*-value for each pairwise comparison

## Discussion

Two goals guided this meta-review, namely, to explore (1) how intervention programs for juvenile offenders influence their return to criminality and (2) how factors of theoretical, practical, and methodological importance impact this relation with recidivism. Main findings from these analyses are discussed next.

There is a significant difference (17.4%) in recidivism between juvenile offenders who do and do not participate in an intervention program. As reflected in this summary effect, programs for juvenile offenders are likely to reduce recidivism and are an important part of offender rehabilitation, reducing recidivism, and enhancing public safety. Despite this encouraging finding, more work is needed to strengthen the positive effects of programs for juvenile offenders. For example, policymakers could leverage this meta-analytic finding to help secure resources to ensure programs are available, equitable, and invested in reentry and wraparound services. Providing such programming may have higher financial costs initially, but not allocating enough resources to properly implement programs to provide consistent support across offender types and criminal justice settings may incur greater long-term societal costs.

While this summary phi coefficient may appear small by conventional benchmarking (Rossi et al., 2019), it is consistent with other effect sizes in the field (Lipsey, 2009; Lipsey & Cullen, 2007). Moreover, the magnitude of this effect is not surprising given discrepant findings across meta-analyses that reinforce our focus on moderators. As expected, the effect size is stronger for some programs and offenders than others, which underscores the need to consider these moderators in theory, research, and practice.

### Take-aways

We conducted five moderator analyses to account for significant variation around the overall effect size, namely type of offender, criminal justice system setting, criminal justice system exposure, program modality, and methodological quality of the included reviews. Although we conducted the moderator analyses two ways, the trends, general magnitude, and relative magnitude of the effect sizes were mostly consistent between the unadjusted and adjusted results. Controlling for publication bias and methodological quality strengthened the effects of certain categories, such as for multisystemic treatment, wilderness therapy, and moderate reviews. However, including these statistical controls attenuated the general magnitude for most of the effect sizes. When accounting for the statistical controls, the statistical significance sometimes changed from the unadjusted to the adjusted effects. For criminal justice system exposure, the magnitudes for diversion and reentry/aftercare were weakened and they both lost statistical significance, although the trends remain the same. Additionally, educational and specialized court program modalities also lost significance in the adjusted models. These differences suggest the necessity of including statistical controls in moderator analyses to reduce potential bias (and potential inflation of effects) that may be attributable to lower quality studies and/or studies that have not undergone the peer-review process. Findings were remarkably consistent with our original hypotheses, with the importance of these findings for researchers, policymakers, and practitioners highlighted next.

#### Offender type

The offender type moderator analysis revealed two main findings. First, participating in an intervention program has the strongest association with a reduction in recidivism for sexual offenders and serious or violent offenders (30.54% and 25.48% difference in recidivism rates, respectively). Second, intervention programs for these two groups produce significantly stronger associations with a reduction in recidivism than for general, nonserious, and drug offenders. Taken together, these findings support that serious offenders have the most opportunity for improvement in their behavior and success upon reentry when participating in a program, suggesting that policymakers and practitioners should include those who are traditionally labeled as “high-risk,” “serious,” or “violent” in early release policies and work-release programs. However, these findings also highlight the necessity for researchers and practitioners to develop more nuanced interventions for less serious and drug offenders. These results may be a function of the best practices developed and tailored for high-risk offenders. As a result, there has been less attention paid to the development of interventions that can best support the needs of lower-risk offenders, which may be due to less research on them or lower fidelity of their implementation. Policymakers should thus consider improvements to current interventions for lower-risk offenders before continuing to swiftly adopt them.

#### Criminal justice setting

The criminal justice setting moderator analysis revealed two main findings. First, participating in an intervention is significantly associated with a reduction in recidivism in both types of criminal justice settings. Second, this effect is nearly three times greater for institutionalized juvenile offenders) than for noninstitutionalized offenders (25.32% and 9.02% difference in recidivism rates, respectively). While the effect is stronger for institutionalized juvenile offenders, it does not negate the significant reduction in recidivism in noninstitutionalized settings. Taken together, correctional researchers and practitioners should be attuned to evaluating and improving community-based correctional programs. Increasing the quality and quantity of treatment services that focus more on support rather than surveillance may provide more positive options for youth and help them avoid the behaviors and environmental factors that may have initially led them to delinquency. It may also be prudent for policymakers to focus on implementing interventions that are cost- and resource-effective that reduce the frequency of juvenile offenders cycling through the system.

#### Criminal justice system exposure

The criminal justice system exposure moderator revealed two main findings. First, only correctional programs had a significant association with a reduction in recidivism (25.40% difference in recidivism). Second, correctional programs are associated with a significantly stronger reduction in recidivism than for diversion and reentry/aftercare programs. Taken together, these results highlight a stark difference between correctional programming, diversion interventions, and reentry/aftercare interventions. Such a difference is likely due to the types of interventions occurring at the diversion or reentry/aftercare level, with the most support and access to programs that induce behavior change implemented for those who are incarcerated. More and better reentry/aftercare programs need to be implemented in a way that does not place youth at greater risk for re-exposure to the justice system, such as programs that help youth secure employment and strengthen connection to the community prior to release. Our findings suggest that juvenile offenders are in most need of reentry services that take a wraparound approach and focus on building relationships and connections that would ultimately provide more opportunities for successful reintegration. Relatedly, diversion programs may not truly divert juvenile offenders, but instead entrench them deeper into the system (see Bouchard & Wong, 2017) while leaving them without the same rehabilitative resources as those formally under correctional supervision. As such, policymakers and practitioners may need to reconsider the widespread implementation of diversion programs in their current form.

#### Program modality

The program modality moderator analysis revealed three main findings. First, program modalities with the strongest outcomes are for multisystemic treatment, family-based treatment, wilderness therapy, cognitive behavioral treatment, and restorative justice (see Table 1)—all of which are significant. Second, multi-systemic treatment was associated with a significantly stronger reduction in recidivism than every other treatment modality. Third, programs that include a rehabilitative component significantly reduce recidivism for juvenile offenders. Taken together, these findings suggest that solely punitive interventions are not enough to enhance public safety and may prove more costly. As such, policymakers should continue allocating funds and resources for programs that are rehabilitative, such as those that include cognitive-behavioral components as well as those that target reflection, accountability, relationship building, and conflict resolution. Researchers and practitioners should also consider that it may not be the program itself driving these differences, but rather the underlying behavior change mechanisms of the programs that may be applicable to more than one program and/or are shared among certain rehabilitative programs. This potential warrants more attention and research on such behavior-change mechanisms as mediating variables along with more collaboration between researchers and practitioners on how to translate the successful intervention components from certain programs into other domains.

#### Methodological quality

The methodological quality moderator analysis revealed two main findings. First, all levels of methodological quality produced significant differences in recidivism, yet low-quality reviews produced the strongest association with a reduction in recidivism (25.18% difference in recidivism). Second, the reduction in recidivism found in low-quality reviews was significantly stronger than found in high-quality reviews. Taken together, these findings underscore the necessity for researchers to adhere to methodological best practices when conducting any review of research because findings from a low-quality review may overestimate the impact of an intervention. As a result, policymakers and practitioners may adopt interventions with inflated effects that are thus less likely to yield the desired or expected result. These possibilities should always be considered when making decisions based on reviews of research.

#### Additional considerations

It is important to note that all the average effect sizes were in the expected direction and in the expected relative magnitude for each moderator. However, it is also important to consider that programs with the strongest effects are not often implemented only for rehabilitative purposes. When considering the potential impact a program may have on recidivism, policymakers and practitioners should consider that the comparison is not simply a rehabilitative model versus a punitive model. While the 40-year debate on what works often emphasizes this dichotomy, program outcomes instead most often reflect a comparison between the punitive model alone and punishment plus rehabilitation. The rehabilitative model is an additive to deterrence-based and accountability strategies (e.g., incarceration or probation), which likely also contributes to the differences observed in the moderator analyses. As such, intervention programs combined with some deterrent value may signal to the youth that even though they are being held accountable for their actions, they still have an opportunity to succeed. When these two conditions (i.e., high accountability and high support) are both present in a program (e.g., via the modality or the context of such program), juvenile offenders may experience the most behavior change as a result of participation.

Indeed, many programs adopted for the most high-risk offenders in the most restrictive circumstances often follow the Risk-Needs-Responsivity (RNR) approach. This approach also helps to explain why institutionalized juvenile offenders, sexual and serious offenders, and incarcerated offenders would have the strongest reductions in recidivism if they participate in a program. Because the RNR model for evidence-based corrections proliferated programs that should target the needs of higher-risk offenders, it is likely that the programs implemented in the last two decades adhere to those best practices. As a result, the effects of more recent programs on recidivism for higher risk offenders may be stronger compared to lower-risk offenders. The programs for higher risk offenders have more research, more targeted responses that are clearly identified, and may be better equipped to induce behavior change.

Moving forward, policymakers and practitioners should consider the importance of addressing more than the needs of juvenile offenders in isolation: it is also salient to preserve remnants of the contexts and lives in which the juvenile will return. Implementing effective programs for juvenile offenders can foster relationships, improve skills, and help juveniles commit to the normative aspects of society (Henggeler et al., 2009). Programs for juvenile offenders can only be effective at reintegration into society if they involve a component of culture and relationships that are representative of their lived experiences (Cullen & Gendreau, 2001). The findings from the last four decades of the “what works” literature are even more urgent in the wake of COVID-19, where the economic impact of the pandemic may raise conversation among agencies, communities, and legislators over the need to reduce or eliminate rehabilitative services. We urge practitioners, legislators, and other policymakers to consider the benefits of rehabilitative approaches and the necessity of implementing programs that include such approaches when making relevant policy decisions moving forward.

#### Limitations

This meta-review provides a unique evaluation of theoretical, practical, and methodological factors that impact the relation between participating in a program and recidivism for juvenile offenders. While the results can inform policy and practitioner decisions about the continued implementation of intervention programs, they should be interpreted in light of four main limitations.

First, it is important to reinforce that the effect sizes we report are averages of averages, which produces a common limitation of meta-reviews. In particular, some effect sizes—stemming from analyses of over 3000 primary studies—may contain inherent bias for a variety of reasons. While the averaging of averages may offset bias present in effect sizes from primary studies, the estimates we calculated are further removed from true effects observed in the world (Polanin et al., 2016). While we can be confident in overall conclusions that suggest intervention programs reduce recidivism for juvenile offenders in different contexts and especially when combined with rehabilitative components, the exact magnitude of these effects cannot be stated with complete confidence (Polanin et al., 2016).

Second, any meta-review relies on the reporting of its included meta-analyses and systematic reviews to be both accurate and complete. In particular, the moderator analyses depend on there being enough data in the included reviews to extract and meaningfully compare across categories of the moderating variable. Moderators can become conflated in a meta-analysis to enable analyses, which in turn influences the way this information can be extracted and coded for a meta-review. Although we were cautious of this possibility when coding and conducting moderators, some moderators may reflect conflation into broader categories. For example, this limitation may be particularly relevant for the program modality moderator, where there is a possibility that modalities may be misclassified in the included reviews and primary studies underlying them. While we coded the modalities based on the authors’ description of programs within each review (Dent, 2015), systematic misclassification stemming from the primary studies (i.e., a program is coded as CBT, but does not actually adopt CBT principles) could account for some differences in the comparable magnitude of category effect sizes.

Third, following from the first two limitations, a moderator analysis on methodological quality of *primary studies* within the included meta-analyses and systematic reviews could not be conducted, which restricts causal inferences. In particular, effect sizes extracted from the included reviews did not typically separate experimental designs from non-experimental designs to compare across them. As a result, we could not disentangle these designs to more confidently estimate a causal effect of intervention programs on recidivism. This more general limitation of research on recidivism could be addressed by incorporating more—and more rigorous—statistical controls in non-experimental designs.

Fourth, most of the phi coefficients presented in this meta-review were modest in magnitude by conventional standards. Yet within empirical context (e.g., Lipsey & Cullen, 2007; Wilson et al., 2018a, b), the magnitude of these effects might have important practical significance in addition to statistical significance. For example, even a 10 or 15% difference in recidivism suggests that there are advantages to intervention programs for some juvenile offenders. When scaled up to the total number of youth involved in the justice system, the actual number of them who may benefit from these programs could be substantial. However, the observed effect sizes may be inflated—a common risk in behavioral science research, as well. For example, the effect sizes in this meta-review would be overestimated if they are in the primary research underlying it (Kim et al., 2013). Selection bias in primary studies that favor treatment groups without randomized assignment could have a trickle-up effect, systematically inflating the effect sizes in this meta-review.

### Future directions

With so many meta-analyses and systematic reviews on intervention programs for juvenile offenders, research calls for a stronger dialogue between primary research and meta-analytic research (Rossi et al., 2019). There needs to be an iterative process by which primary research authors engage with respective meta-analyses to use them as a tool for advancing the growth of knowledge within the field. Meta-analysis (and meta-reviews) acts as a survey of the empirical landscape, which can help identify gaps in primary research that need to be filled to clarify and extend our empirical understanding of programs for juvenile offenders. For example, researchers might consider more evaluations on programs studied less often, such as those used in reentry/aftercare services, to identify additional contexts that might moderate treatment effects for juvenile offenders.

Primary research and meta-analytic research on intervention programs for juvenile offenders have primarily emphasized the outcome of recidivism. When focusing on this outcome, authors of both primary research and meta-analyses should consider including the definition of recidivism more often as a moderator to examine how the effects of programming may differ as a function of self-report, technical violations, new arrests, and new convictions. Relatedly, few meta-analyses include other outcomes in addition to recidivism because primary studies often do not report other outcomes. Intervention programs for juvenile offenders should continue to be evaluated as programs develop and adapt over time. However, it is important for future primary research to include other measures of success alongside recidivism as to assess the effect of intervention programs on them. Additional predictive factors should also be included in future primary research that captures the social ecological context of juvenile offenders, such as social economic status, location of reintegration, level of exposure to violence, family support, and religiosity. These additional factors can then situate the effectiveness of intervention programs within context to reflect the lived experience of justice-involved youth.

Given the increasing number of meta-reviews, greater attention on the methodological quality and reporting of meta-analyses is a valuable goal of cumulative science. Many best practices currently exist for reporting data in a meta-analysis (e.g., PRISMA, Page et al., 2021; MARS, American Psychological Association, 2008), which authors use to ensure transparent, standardized, and accurate coding and reporting. Yet meta-analysts should also refer to the standards for inclusion in a meta-review, such as AMSTAR-2 (e.g., Shea et al., 2017), GRADE (e.g., Guyatt et al., 2008), or the EMMIE framework (e.g., Johnson et al., 2015), given the possibility that their meta-analyses may be included in future meta-review(s). For example, systematic reviews and meta-analyses are often longer than primary studies and concern over page length may require omission of detailed information regarding coding decisions. One solution is to take full advantage of online supplements to include more detailed justifications of decisions along with information about the coding and combining of categories for analysis. Just as it is necessary for certain statistical information to be present in a primary study to generate effect sizes for a meta-analysis, the same is true about meta-analyses in a meta-review. Although meta-analyses already report average effect sizes, confidence intervals or standard errors should more consistently accompany them especially for moderator analyses. Additionally, meta-analysts can leverage space using a summary table to provide detailed information coded from each included primary study. We urge meta-analysts to consider adopting these transparent reporting and recoding decisions given their influence on policy decisions (Polanin et al., 2016). Likewise, authors of future meta-reviews will depend on that transparent reporting to conduct more advanced meta-review analyses and ultimately more impactful research (Polanin et al., 2020).

## Conclusion

The “what works” question has dominated discourse and research on intervention programs for juvenile offenders across four decades, sustaining the attention of policymakers and practitioners (Lipsey & Cullen, 2007). These programs are a crucial component of assessing the juvenile justice system in its role to rehabilitate youth and allocate funding for programs that work best. This meta-review explored the association between participating in an intervention program and recidivism for juvenile offenders based on four decades of primary and secondary research. Our analyses revealed that intervention programs are associated with a significant reduction in recidivism for juvenile offenders, suggesting that the rehabilitative model is more promising in this regard than the punitive model alone. Overall, programs that target a response to the micro- and meso-level needs of the offender (e.g., multisystemic treatment, family-based treatment) combining rehabilitative and deterrence-based strategies show the strongest impact on recidivism for juvenile offenders. With this integrative information, policymakers and practitioners can more confidently work toward creating a criminal justice system that balances holding a juvenile accountable for their actions while still fostering their ability to reintegrate into society.

## Supplementary information

ESM 1 (PDF 142 kb)

ESM 2 (PDF 392 kb)

ESM 3 (PDF 107 kb)

## Footnotes

1 In this meta-review, “juvenile offender” refers to youth who are involved in the justice system. The term “youth” refers to youth who are not involved in the justice system.

2 In this meta-review, “intervention” refers to rehabilitation programs, treatment programs, and other forms of interventions used with juvenile offenders along the spectrum of justice system involvement. In this meta-review, “intervention” does not refer to programs used with youth not involved in the justice system, which would otherwise be labeled as prevention programs, rather than intervention programs.

3 When we refer to the word “review” by itself, we mean secondary research in the form of meta-analyses and systematic reviews, rather than tertiary research in the form of a meta-review.

4 PICOS refers to Participant, Intervention, Comparison, Outcome, and Study Design (Uman, 2011) and is the process for determining the criteria that will guide which studies are selected for the meta-analysis or meta-review.

5 One meta-analysis (Petrosino et al., 2010) compared formally processed juvenile offenders with juvenile offenders who experienced diversion. This meta-analysis was retained despite its flipped comparison group because the information provided was deemed valuable and necessary for inclusion in this meta-review, with its effect size (Cohen’s *d*-index) easily transformed to maintain its magnitude. We transformed the effect sizes, such that the directionality of the effect would represent the juvenile offenders experiencing diversion as the treatment group and the formally processed juvenile offenders as the comparison group.

6 We initially retrieved reports that included other outcomes, but there were not enough of these additional outcomes (beyond recidivism) to meaningfully and appropriately incorporate them into this meta-review.

7 Two meta-analyses (Livingstone et al., 2013; Reitzel & Carbonell, 2006) included 7-year old juveniles, but most of their participants were over the age of 10, so these two studies were included in this meta-review.

8 Cohen’s *d* effect sizes converted to phi as phi = d/√(4 + d)^2^; odds ratios converted to *d* as *d* = Log(OR)/2, then *d* converted to phi (Lipsey & Cullen, 2007). Authors were contacted when necessary to gather information relevant to the studies that were included in any meta-analyses or systematic reviews when needed to calculate relevant effect size for this meta-review. However, those correspondences produced additional data for 10 meta-analyses, but for no systematic reviews.

9 We initially attempted to gather all of the primary studies that appear in qualifying reviews of research in order to identify and de-duplicate studies that appear in multiple reviews. Given the prohibitive barriers to doing so (e.g., meta-analyses not providing a list of included primary studies, authors no longer maintaining the information, authors no longer alive, lack of access to full versions of primary studies), collecting this entire population of studies was impossible. The resulting absence of primary studies would thus undermine conclusions more than potential overlapping of them across different reviews of research. The complete rational for this decision is provided in the Supplemental File.

10 This meta-review uses a phi coefficient, which is based on a Pearson’s product moment correlation coefficient. A Fisher transformation was calculated for the same reason one is needed for a traditional correlation coefficient. This transformation converts the phi or correlation coefficient into a *z*-score to stabilize the variance and create a normal sampling distribution, allowing a confidence interval to be constructed around the parameter estimate that is equidistant to the upper and lower bounds (Cohen et al., 2002; Cooper, 2010). After confidence intervals were calculated, the confidence intervals and their average effect sizes were converted back into phi coefficients.

11 Given the large number of program names studied across all of the included reviews and the overlap in their underlying principles, we chose to use those underlying principles to categorize effect sizes by their program modalities to enable more meaningful and consistent analyses (Dent,  2015). For program modality, we distinguished diversion programs from restorative justice programs to isolate the differences between modalities that were identified as restorative from programs that simply aimed to divert offenders either through caution programs or police-initiated programs. Restorative justice has principles unique to its implementation that made it necessary to separate and explore its nuanced effects.
