# Supplementary material for: The 40-year debate: a meta-review on what works for juvenile offenders
Source: J Exp Criminol. 2021 Jun 12;19(1):1–30. doi: 10.1007/s11292-021-09472-z (PMC8196268; doi:10.1007/s11292-021-09472-z)
Supplement: Supplementary file 1 — (PDF 142 kb) [file 11292_2021_9472_MOESM1_ESM.pdf]

SUPPLEMENTAL FILE A

Hypothesis justification, pilot-testing procedures, and methodological justifications.

MODERATOR HYPOTHESES

Type of Offender

Programs for serious or violent offenders and sexual offenders are expected to have the strongest association with a reduction in recidivism.[1] Based on the RNR framework, if programs targeting juvenile offenders who have committed serious or violent crimes are more attentive to the needs of those offenders as a model of change, then there should be a greater reduction in recidivism than for non-serious or non-violent offenders (Dowden & Andrews, 2000). Similarly, programs for drug offenders should show a stronger association with a reduction in recidivism than for non-serious or non-violent offenders, but weaker than for serious and sexual juvenile offenders. If programs for drug offenders respond to the unique needs of this group, then they should benefit more than non-serious offenders, but not as much as serious offenders due to a difference in risk level (Bouchard & Wong, 2017). If a program does not adhere to RNR best practices and instead combines serious offenders with non-serious offenders, then these juvenile offenders could learn behaviors and techniques from each other, which may contribute to continued engagement in delinquent behavior (Bernburg et al., 2006). As a result, programs for non-serious and non-violent juvenile offenders are expected to have the weakest association with a reduction in recidivism.

Criminal Justice Setting

Treating a juvenile offender in an institutionalized setting is expected to show the strongest association with a reduction in recidivism. Most of the programs included in the meta-

[1] We adopt the phrase "weaker/stronger association with a reduction in recidivism" throughout these sections because that is how the effect is quantified in this meta-review.

analytic literature focus on the RNR model of treatment and, as such, are likely to influence the effect of programs on recidivism. If institutionalized juvenile offenders are treated with programs tailored to their risks and needs, such responsiveness may complement the deterrent value of institutionalization. As a result, institutionalized juvenile offenders are likely to experience the benefits of these programs more strongly than juvenile offenders in a noninstitutionalized setting. While treating juvenile offenders in a noninstitutionalized setting is also expected to show an association with a reduction in recidivism, this relation is expected to be weaker than for institutionalized offenders. A noninstitutionalized setting may decrease labeling effects, limit the criminogenic effect, and exposure to the criminal justice system may deter juvenile offenders from engaging in criminal behavior again. However, these programs may be less targeted and may lead these youth to greater law enforcement surveillance (e.g., Petersilia & Turner, 1993), which may weaken the strength of the program's effect on recidivism.

Criminal Justice System Exposure

Diversion programs are expected to have a weaker association with a reduction in recidivism than programs for incarcerated youth, but a stronger association than for reentry and aftercare programs. Juvenile offenders should benefit from diversion programs if they reduce the stigma of being formally adjudicated (Wilson et al., 2018a). Diversion programs often operate under a labeling theory framework (Bouchard & Wong, 2017; Butts et al., 2002), which may be responsible for these programs showing a greater reduction in recidivism for juvenile offenders when compared to traditional processing or treatment as usual (e.g., Wilson & Hoge, 2013). However, this reduction in recidivism may be weaker than programs for incarcerated youth. Diversion programs often target low-risk juvenile offenders, whereas incarceration is reserved for higher risk juvenile offenders (Wood & Sazuki, 2016). If programs adhere to best practices

and target the needs of offenders, the positive impact of the RNR model in programs might outweigh the negative impact of labeling and result in programs for incarcerated juveniles having the strongest association with a reduction in recidivism.

Programs for juvenile offenders in re-entry or aftercare services are expected to have the weakest association with a reduction in recidivism. Some of the offenders in these aftercare services may be transitioning from correctional facilities and participating in services that involve community supervision among other service provisions (e.g., Weaver & Campbell, 2015). If these services ultimately continue to expose a juvenile offender to the criminal justice system, then they are still susceptible to the negative effect of stigmatization as a function of continuous surveillance by law enforcement and are more likely to be rearrested for technical violations (Bouchard & Wong, 2018). Therefore, reentry and aftercare services are expected to have the weakest association with a reduction in recidivism compared to programs implemented for diverted or incarcerated juvenile offenders.

Program Modality

Family-based interventions and MST are expected to show the strongest associations with a reduction in recidivism due to their focus on maintaining family connections and relationships with those that are crucial to their success (Henggeler et al., 2009). If these programs preserve these values so that the stigma of being labeled an offender can be reduced, then juvenile offenders in them may experience a greater reduction in recidivism after contact with the juvenile justice system. Relatedly, while CBT program modalities consistently produce some of the strongest and most beneficial outcomes for offenders (Lipsey et al., 2007), they do not necessarily account for the other people in the lives of youth who might model either

negative or positive behavior (Henggeler et al., 2009). As a result, CBT programs are expected to show a weaker association than family interventions and multi-systemic treatment.

Restorative justice programs operate under a reintegrative shaming model that encourages accountability and restoration of relationships (Strang et al., 2013). However, some evidence shows that they reduce recidivism for juvenile offenders (e.g., Bradshaw et al., 2006), while others show nonsignificant or small effects (e.g., Livingstone et al., 2013). Given these inconsistent findings, restorative justice programs are expected to have a weaker association with a reduction in recidivism for juvenile offenders than other treatment modalities. Similarly, specialized courts and other diversion programs are also expected to show a weaker association with a reduction in recidivism. While these programs aim to prevent the stigmatizing and labeling effect associated with being formally adjudicated (Bernburg et al., 2006), they still target low-risk juvenile offenders and do not specifically address behavior change. Comparatively, these programs may not have as strong of an effect as programs that formally integrate behavior change components.

Deterrence-based programs such as shock incarceration, scared straight, and boot camps are expected to show the weakest association with a reduction in recidivism. Deterrence-based programs should prevent future delinquency by exposing a juvenile offender to the harsh consequences of punitive discipline. For example, proponents of intensive supervision probation suggest that frequent contacts between probation and the offender prevent those youth from committing more crime (Bouchard & Wong, 2018). However, those frequent contacts with law enforcement function as a net-widening mechanism, such that juvenile offenders may be further introduced to the system and receive more technical violations. As such, this unintended effect may result in the weakest associations with a reduction in recidivism.

Methodological Quality

Low-quality systematic reviews and meta-analyses are expected to show the strongest association with reductions in recidivism for juvenile offenders participating in intervention programs (Shea et al., 2009). Moderate- and high-quality reviews likely adhere more closely to best practices and better mitigate bias, preventing effect sizes from becoming artificially inflated. Therefore, high-quality reviews are expected to have the weakest association with reduced recidivism.

METHOD

Process for Generating Search Terms

The first strategy was comprehensively searching electronic databases (between July and September of 2018) with terms generated through an iterative and innovative process. After examining the journal coverage of major databases with a sample of meta-analyses found through Google Scholar web searches, we searched ten electronic databases: *PsycINFO, ERIC, Sociological Abstracts, ProQuest Dissertation Abstracts and Theses, Criminal Justice Abstracts, JSTOR, RAND Corporation, and Campbell Collaboration, Academic Search Complete, National Criminal Justice Research Service (NCJRS), and the Office Of Juvenile Justice Delinquency and Prevention (OJJDP).* We generated this list to reduce the number of duplicate reports across the databases as well as to increase the chance of gathering reports considered “grey literature” that may not be published through traditional academic channels.

In order to search these electronic databases, we generated and pilot tested a list of search terms to make sure all relevant reviews would be captured by the search. Some of the search terms were generated by previous meta-analyses (Schwalbe et al., 2011; James et al., 2012; Weaver & Campbell, 2015; Bouchard & Wong, 2018), some of them were inspired by key words

of related studies (Bouffard & Bergseth, 2008; Lipsey, 2009; Abrams & Snyder, 2010; Lambie & Randell, 2013), and some of them were generated independently by the authors. After pilot testing these terms, the literature search was conducted for all databases on July 2, 2018 using the "everywhere but full text" option. This search retrieved 1,596 documents across five ProQuest databases: PsychINFO, ERIC, Sociological Abstracts, Dissertation Abstracts and Theses, and Criminal Justice Abstracts. The EBSCO database Academic Search Complete returned 87 results, and JSTOR returned 304 search results. The remaining databases had modified search terms due to constrictions by the databases on the number of search terms. RAND Corporation returned 82 search results, Campbell Systematic Reviews returned 35 search results, NCJRS returned 52 search results, and OJJDP returned 4 search results. Taken together, these ten electronic databases collected 2,160 documents (see Supplemental Table A).

Process for Developing Full-Text Screening Guide

We developed the full-text screening guide by taking a sample of three articles that should be included in the meta-review and screening them in their entirety based on the a priori inclusion and exclusion criteria specified below. Next, we pilot tested the full-text screening guide on a separate sample of three articles that should be included in the meta-review to check that it would not exclude any of those articles based on the tool's questions (see Appendix B).

Inclusion Criteria Justification

First, we retained meta-analyses and systematic reviews if they met the qualifying criteria for a systematic review (O'Connell et al, 2013) determined by the updated AMSTAR-2 tool (Shea et al., 2017). Item four of AMSTAR-2 provides the criteria for a comprehensive literature search strategy, and any review of research that could answer at least "partial yes" was included in the meta-review (if it also met the substantive criteria). Second, meta-analyses and systematic

reviews that focused exclusively on juvenile offenders were retained. The age range for juvenile offenders varies across studies (e.g., Lipsey, 2009; James et al., 2012), so meta-analyses and systematic reviews were included in this meta-review if they integrated primary studies of juvenile offenders aged 10 to 25 to maximize the number of studies available and to account for juvenile correctional facilities that house offenders of older ages. Third, we included meta-analyses and systematic reviews that examined any program as long as the juvenile had made contact with the criminal justice system to enable comparison across a wide range of programs. Fourth, meta-analyses and systematic reviews must have examined interventions that had a treatment condition where the juvenile offender received a type of program and a comparison condition where the juvenile offender did not experience that type of program. Fifth, this meta-review includes meta-analyses and systematic reviews that measured recidivism broadly, including new contact with law enforcement, rearrests, reconviction, and/or probation violations. Most meta-analyses and systematic reviews on the effectiveness of programs for juvenile offenders use recidivism as the criminality outcome measure; however, the definition of recidivism differs across states and studies (Bird & Grattet, 2016). Sixth, only meta-analyses and systematic reviews that are reported in English were included in this meta-review to ensure that no information was missed or interpreted incorrectly.

Coding Pilot Tests and Response Option Rational

Additional steps were taken to fully capture all relevant information needed to conduct the moderator analyses. First, we collected a random subset of known meta-analyses that met the inclusion criteria. All relevant coding variables that appeared in those studies were written onto a coding sheet along with other variables generated outside of those studies that were also deemed relevant. Next, we gathered another random subset of meta-analyses and pilot tested the working

coding protocol on them. This coding process helped ensure that relevant information appearing in meta-analyses and systematic reviews was not missed, otherwise potential moderator analyses would not be able to be conducted. This procedure also helped account for different ways that meta-analyses and systematic reviews report their data, so that a template of how to code them could be provided in the protocol to promote consistency in coding.

Codes were classified into one or more predefined categories, including “Other” and “Varied” options. For both the “Other” and “Varied” categories, there was the option to specify why the review could not be classified in any of the pre-specified categories. These two additional categories allow for each variable coded to be comprehensive and mutually exclusive (Cooper, 2010) and to prevent the coder from choosing a pre-specified category that may not fully represent the coded information. Some codes were more likely to involve subjective judgment or lead to more discrepancies. In these cases, the coding protocol called for evidence from the original work in the authors’ own words when possible to support resolving coder discrepancies. The final coding protocol can be found in the supplemental file.

Identifying Independent Samples Rational from Footnote 9

One anonymous reviewer raised a concern over possible overlap among meta-analyses and the potential for a primary study to be included more than once in the meta-review as a result. While we agree there could be some overlap in primary studies across the meta-analyses, there is unfortunately no way to quantify its amount given that many meta-analyses do not offer a listing of the studies included in them. However, there is compelling reason to believe potential overlap is not impacting the results we – and likely stakeholders – value most: moderators. While redundant studies may contribute to the summary effect, we expect much less – if any – in the moderator analyses. Given that the focus of this meta-review is the moderators and not the

summary effect, we would be most concerned about redundancy of primary studies underlying the moderator categories. However, we do not suspect this issue is at play for the two main reasons. First, we expect that most of the potential overlap, if any, would exist in the older studies before there was a large body of research focusing on the nuanced areas of correctional programming as well as its subfields (e.g., drug courts, reentry services). Since these newer meta-analyses are looking at different — and more specific — areas of correctional programming, there is much less chance for overlap of the primary studies.

Second, we took steps to enhance the integrity and clarity of moderator categories in an effort to reduce potential redundancy of studies within them. This strategy involved removing the effect sizes from certain moderator analyses if their category could not be clearly coded or if the effect size potentially reflected more than one category. In the program modality moderator, for example, some meta-analyses examined programs broadly that may have led to overlap between those meta-analyses and others that adopted a similarly broad approach. If their effect sizes did not distinguish CBT programs from Restorative Justice, for example, they were coded as "Varied" and removed from the moderator analysis due to concerns about clarity and redundancy. As such, the moderator analyses presented in our meta-review have already been addressed for that potential overlap, while still retaining as much of the data as possible. Because many meta-analyses included in this meta-review do not provide a list of their primary studies, if we switched to them as the unit of analysis, we would lose many studies that underlie the results of the meta-review and uniquely allow us to address its core questions.

Third, we included methodological controls in our meta-regression models to reduce bias potentially arising from reviews that were not peer-reviewed and/or of low methodological quality. As part of this methodological quality assessment, reviews of high methodological

quality were ones that accounted for bias and outliers in their own analyses (e.g., through winsorizing their outliers or conducting sensitivity analyses). As a result, the adjusted effect sizes in our meta-review reflected meta-analyses of the highest quality that themselves addressed outlying studies. These adjusted effect sizes were not meaningfully different, either statistically or substantively, from the unadjusted effect sizes. Taken together, for these three reasons, we can be reasonably confident that redundant and especially outlying studies are not swaying the results of moderator analyses, which are the primary focus of this meta-review.

REFERENCES

Abrams, L. S. & Snyder, S. M. (2010). Youth offender reentry: Models for intervention and directions for future inquiry. *Children and Youth Services Review, 32*(12), 1787-1795.

Bird, M. & Grattet, R. (2016). Realignment and recidivism. *The ANNALS of the American Academy of Political and Social Science, 664*(1), 167-195.

Bernburg, J. G., Krohn, M. D., & Rivera, C. J. (2006). Official labeling, criminal embeddedness, and subsequent delinquency: A longitudinal test of labeling theory. Journal of Research in Crime and Delinquency, 43(1), 67-88.

Bouchard, J., & Wong, J. S. (2017). A Jury of Their Peers: A meta-analysis of the effects of teen court on criminal recidivism. Journal of Youth and Adolescence, 46(7), 1472-1487.

Bouchard, J. & Wong, J. S. (2018). Examining the effects of intensive supervision and aftercare programs for at-risk youth: A systematic review and meta-analysis. *International Journal of Offender Therapy and Comparative Criminology, 62*(6), 1509-1534.

Bouffard, J. A. & Bergseth, K. J. (2008). The impact of reentry services on juvenile offenders' recidivism. *Youth Violence and Juvenile Justice, 6(3),* 295-318.

Bradshaw, W., Roseborough, D., & Umbreit, M. S. (2006). The effect of victim offender mediation on juvenile offender recidivism: A meta-analysis. Conflict Resolution Quarterly, 24(1), 87-98.

Butts, J., Buck, J., & Coggeshall, M. (2002). The impact of Teen Court on young offenders: Research report. Urban Institute Justice Policy Center.

Cooper, H. (2010). *Research synthesis and meta-analysis: A step-by-step approach*. Thousand Oaks: SAGE.

Henggeler, S. W., Schoenwald, S. K., Borduin, C. M., Rowland, M. D., & Cunningham, P. B. (2009). Multisystemic therapy for antisocial behavior in children and adolescents. New, York, NY: The Guilford Press.

James, C., Stams, G.J., Asscher, J.J., Katrien De Roo, A., & Van der Laan, P.H. (2013). Aftercare programs for reducing recidivism among juvenile and young adult offenders: A meta-analytic review. *Clinical Psychology Review, 33*(2), 263-274.

Lambie, I. & Randell, I. (2013). The impact of incarceration on juvenile offenders. *Clinical Psychology Review, 33*(3), 448-459.

Lipsey, M.W. (2009). The primary factors that characterize effective interventions with juvenile offenders: A meta-analytic overview. *Victims and Offenders, 4*(2), 124–147.

Lipsey, M.W., Landenberger, N.A., & Wilson, S.J. (2007). Effects of cognitive-behavioral programs for criminal offenders. Campbell Systematic Reviews, 2007(6), 1-27.

Livingstone, N., Macdonald, G., & Carr, N. (2013). Restorative justice conferencing for reducing recidivism in young offenders (aged 7 to 21). Cochrane Database of Systematic Reviews, 2013(2), 1-89.

O'Connell, N.E., Wand, B.M., McAuley, J., Marston, L., & Moseley, G.L. (2013). Interventions for treating pain and disability in adults with complex regional pain syndrome- an overview of systematic reviews. *Cochrane Database of Systematic Reviews, 2013*(4), 1-70.

Petersilia, J., & Turner, S. (1993). Intensive probation and parole. Crime & Justice, 17, 281-335.

Schwalbe, C. S., Gearing, R. E., MacKenzie, M. J., Brewer, K. B., & Ibrahim, R. (2012). A meta-analysis of experimental studies of diversion programs for juvenile offenders. *Clinical Psychology Review, 32*(1), 26–33.

Shea, B. J., Hamel, C., Wells, G. A., Bouter, L. M., Kristjansson, E., Grimshaw, J., Henry, D. A., & Boers, M. (2009). AMSTAR is a reliable and valid measurement tool to assess the methodological quality of systematic reviews. Journal of Clinical Epidemiology, 62(10), 1013-1020.

Shea, B.J., Reeves, B.C., Wells, G., Thuku, M., Hamel, C., Moran, J., Moher, D., Tugwell, P., Welch, V., Kristjansson, E., & Henry, D.A. (2017). AMSTAR 2: a critical appraisal tool for systematic reviews that include randomised or non-randomised studies of healthcare interventions, or both. *BMJ*.

Strang, H., Sherman, L. W., Mayo-Wilson, E., Woods, D., & Ariel, B. (2013). Restorative justice conferencing (RJC) using face-to-face meetings of offenders and victims: Effects on offender recidivism and victim satisfaction—A systematic review. Campbell Systematic Reviews, 2013(12), 1-59.

Weaver, R.D. & Campbell, D. (2014). Fresh start: A meta-analysis of aftercare programs for juvenile offenders. *Research on Social Work Practice, 25(2),* 201-212.

Wilson, D. B., Brennan, I., & Olaghere, A. (2018a). Police-initiated diversion for youth to prevent future delinquent behavior: A systematic review. Campbell Systematic Reviews. 2018(5), 1-85.

Wilson, H. A., & Hoge, R. D. (2013). The effect of youth diversion programs on recidivism. Criminal Justice and Behavior, 40(5), 497-518.

Wood, W.R. & Suzuki, M. (2016): Four challenges in the future of restorative justice. Victims & Offenders, 11(1), 149-172.
