# Supplementary material for: The 40-year debate: a meta-review on what works for juvenile offenders
Source: J Exp Criminol. 2021 Jun 12;19(1):1–30. doi: 10.1007/s11292-021-09472-z (PMC8196268; doi:10.1007/s11292-021-09472-z)
Supplement: Supplementary file 2 — (PDF 392 kb) [file 11292_2021_9472_MOESM2_ESM.pdf]

SUPPLEMENTAL FILE B

Tables and tools referred to throughout the manuscript.

SUPPLEMENTAL TABLE A Summary of hypotheses for the theoretical and methodological moderator

Moderator	Hypothesis
Offender Type	Serious juvenile offenders and sexual juvenile offenders are expected to have the strongest associations with reducing recidivism.<br>Drug offenders are expected to have a weaker correlation with reducing recidivism than serious or sexual offenders, but a stronger association with reducing recidivism than non-serious and non-violent offenders.
Criminal Justice Setting	Institutionalized juvenile offenders are expected to have stronger associations with a reduction in recidivism than non-institutionalized juvenile offenders.
Criminal Justice System Exposure	Correctional programs are expected to have the strongest associations with a reduction in recidivism.<br>Diversion programs are expected to have stronger associations with a reduction in recidivism than for reentry/aftercare programs, but weaker than correctional programs.
Type of Program Modality	Family-based interventions, multi-systemic treatments, and cognitive behavior programs are expected to have the strongest associations with a reduction in recidivism.<br>Educational programs, restorative justice programs, and wilderness programs are expected to have stronger associations with a reduction in recidivism than specialized courts and other diversion programs, but weaker than family-based, multi-systemic, and cognitive-behavior interventions.<br>Intensive supervision probation and shock incarceration are expected to have the weakest associations with a reduction in recidivism.
Methodological Quality	Low-quality reviews are expected to have the strongest associations with a reduction in recidivism.<br>Moderate-quality reviews are expected to have a stronger association with a reduction in recidivism than high-quality reviews, but a weaker association than low-quality reviews.

SUPPLEMENTAL TABLE B Overview of literature search parameters for electronic databases

Literature Search	Search Terms	Search Parameters	Electronic Databases	Documents Retrieved
Terms	"juvenile offender*" or "juvenile delinquent*" or "youth offender*" or "youth delinquent*" or "juvenile and young adult offender*" AND program* or intervention* or rehab* or therap* or diversion* or aftercare or reentry or prevent* reintegrat* or incarcer* or alternative* or "restorative justice" or restore* or "victim offender mediation" or "community treatment" or "cognitive behavior therapy" or treatment or education* or vocation* or service* AND "meta analy*" or review	The abstract of documents was searched using the ProQuest and EBSCO search engines.	*PsycINFO, ERIC, Sociological Abstracts, ProQuest Dissertations and Theses (2), Criminal Justice Abstracts, Academic Search Complete*	1683
	"juvenile offender*" or "juvenile delinquen*" AND program* AND "meta analy*" or "systematic review" or therap* or rehab* or intervent* or prevent* AND "meta analy*" or "systematic* review"	The abstract of documents was searched using the JSTOR and search engines.	*JSTOR*	304
	"juvenile"	The abstract of documents was searched using the RAND Corporation and NCJRS search engines.	*RAND, NCJRS*	134
	"youth"	The abstract of documents was searched using the Campbell Systematic Review search engine.	*Campbell Systematic Reviews*	35
	"systematic review"	The abstract of documents was searched OJJDP search engine.	*OJJDP*	4

Note. "meta-analy*" was chosen instead of "meta-analysis" to capture studies that used different variations of that word in their abstract.

SUPPLEMENTAL TABLE C Information coded in research reports

**Report Characteristics**
1. Author name
2. Year of publication
3. Report type (e.g., journal article, book chapter, dissertation, etc.)
4. Review type
5. Literature inclusion
Setting Characteristics
1. Location of primary studies
2. Location of authors of review
3. Criminal justice setting (e.g., institutional and non-institutional)
4. Type of institution (public or private)
Participant (study) Characteristics
1. Sample identification number
2. Defining characteristics of the sample
3. Subgroup analysis
4. Socioeconomic status
5. Average age of juvenile offenders
6. Median Age of juvenile offenders
7. Age range of juvenile offenders
8. Number of studies included in the review
9. Ratio of males to females
10. Race/ethnicity breakdown and ratio
11. Delinquency level
12. Offense history
Intervention Information
1. Type of intervention/program being studied (e.g., diversion, correctional, reentry/aftercare)
2. Timing of the intervention/program (e.g., before incarceration, during incarceration, after incarceration)
3. Researcher Involvement
4. Type of methodological design
5. Type of comparison group
6. Fidelity of implementation measurement
7. Dosage information
8. Evidence-based program
Outcome Information
1. Outcome ID number
2. Type of outcome measure being reported (e.g., recidivism, educational attainment)
3. Operationalization of outcome measure
4. Follow-up period of outcome measure
Effect Size Information
1. Effect size index
2. Effect size
3. Confidence intervals
4. Model type
5. Heterogeneity measure

SUPPLEMENTAL TABLE D Table of excluded studies

Author(s)	Year	Reason for Exclusion
Menon & Cheung	2018	Not measuring an effect of an intervention or treatment program.
Branson & Baetz	2017	Not measuring an effect of an intervention or treatment program.
Black	2016	Not measuring an effect of an intervention or treatment program.
Gonzales	2014	Not a meta-analysis or a systematic review.
Welsh & Rocque	2014	An overview of reviews.
Benuto	2014	Not a meta-analysis or a systematic review.
Ferguson & Wormith	2012	Not focusing on juvenile offenders.
Ioane et al.	2013	Not a meta-analysis or a systematic review.
Foy et al.	2012	Not a meta-analysis or a systematic review.
Tripodi & Bender	2011	No relevant outcomes.
Meade & Steiner	2010	Not enough extractable data.
Klenowski et al.	2010	Not enough extractable data.
Marshall & Burton	2010	Not a meta-analysis or a systematic review.
Quinn & Shera	2009	Not a meta-analysis or a systematic review.
Weis & Toolis	2008	Not a meta-analysis or a systematic review.
Elwyn & Ryst	2007	Not measuring an effect of an intervention or treatment program.
Bala et al.	2009	Not a meta-analysis or a systematic review.
Borduin & Schaeffer	2008	Not enough extractable data.
West & Crompton	2001	Not a meta-analysis or a systematic review.
Richards & Sullivan	1996	Not a meta-analysis or a systematic review.
Basta & Davidson	1988	Not measuring an effect of an intervention or treatment program.
Pusch	2016	Not measuring an effect of an intervention or treatment program.
Mularski	2006	Not a meta-analysis or a systematic review.
Redding	2003	Not measuring an effect of an intervention or treatment program.
Cramer	2004	Not a meta-analysis or a systematic review.
Cervantes	2013	Not a meta-analysis or a systematic review.
Underwood & Knight	2006	Not a meta-analysis or a systematic review.
Borduin & Schaeffer	2002	Not a meta-analysis or a systematic review.
Umbreit et al.	2001	Not a meta-analysis or a systematic review.
Roget et al.	1998	Not measuring an effect of an intervention or treatment program.
Steiner & Cauffman	1998	Not measuring an effect of an intervention or treatment program.
Snyder	1996	Not measuring an effect of an intervention or treatment program.
Henggeler et al.	1994	Not enough extractable data.
Fagan	1991	Not a meta-analysis or a systematic review.
Goldsmith	1987	Not measuring an effect of an intervention or treatment program.
Piper & Warner	1982	Not a meta-analysis or a systematic review.
Feldman et al.	1973	Not a meta-analysis or a systematic review.
Adamson & Dunham	1956	Not a meta-analysis or a systematic review.
Petrosino	1997	Not focusing on juvenile offenders.
Letourneau et al.	2017	Not focusing on juvenile offenders.
Sprott & Manson	2017	Not a meta-analysis or a systematic review.
Kim et al.	2015	An overview of reviews.
MacKenzie & Farrington	2015	An overview of reviews.
Hong et al.	2015	No relevant outcomes.
Manchak & Cullen	2015	Not a meta-analysis or a systematic review.
May et al.	2014	Not measuring an effect of an intervention or treatment program.
Creaney	2014	Not measuring an effect of an intervention or treatment program.

Matz	2014	Not measuring an effect of an intervention or treatment program.
Evans-Chase & Zhou	2012	Not enough extractable data.
Bain	2012	Not focusing on juvenile offenders.
Buston et al.	2012	No relevant outcomes.
Sells et al.	2012	Not measuring an effect of an intervention or treatment program.
Mitchell et al.	2012a	Not focusing on juvenile offenders.
Van Vugt et al.	2011	Not measuring an effect of an intervention or treatment program.
Penner et al.	2011	Not a meta-analysis or a systematic review.
Caldwell	2010	Not measuring an effect of an intervention or treatment program.
Townsend et al.	2010	No relevant outcomes.
Riphagen	2010	Not enough extractable data.
Zahn et al.	2009	Not enough extractable data.
Underwood et al.	2008	Not enough extractable data.
Van Lier et al.	2007	No relevant outcomes.
Hayes	2006	Not a meta-analysis or a systematic review.
Andrews & Dowden	2006	Not focusing on juvenile offenders.
Landenberger & Lipsey	2005	Not focusing on juvenile offenders.
Umbreit et al.	2004	Not a meta-analysis or a systematic review.
Curtis et al.	2004	Not focusing on juvenile offenders.
Littell	2005	Not enough extractable data.
Littell et al.	2005	Not focusing on juvenile offenders.
Cameron & Telfer	2004	Not measuring an effect of an intervention or treatment program.
Spencer & Jones-Walker	2004	Not a meta-analysis or a systematic review.
Borum	2003	Not a meta-analysis or a systematic review.
Tarolla et al.	2002	Not enough extractable data.
Perkins-Dock	2001	Not a meta-analysis or a systematic review.
Nugent et al.	2001	Not a meta-analysis or a systematic review.
Ertl & McNamara	1997	Not measuring an effect of an intervention or treatment program.
Losel	1995	An overview of reviews.
Van Nagel et al.	1986	Not enough extractable data.
Tolan et al.	1986	Not a meta-analysis or a systematic review.
Henderson & Hollin	1983	Not enough extractable data.
Peterson et al.	1976	Not measuring an effect of an intervention or treatment program.
Matjasko et al.	2012	An overview of reviews.
MacKenzie et al.	2001	Not focusing on juvenile offenders.
Wilson et al.	2005	Not focusing on juvenile offenders.
Lipsey et al.	2007	Not focusing on juvenile offenders.
Mitchell et al.	2012b	Not focusing on juvenile offenders.
Davis et al.	2014	Not enough extractable data.
Mitchell et al.	2006	Not focusing on juvenile offenders.
McGuire	2008	An overview of reviews.
Latessa	2006b	An overview of reviews.
Latessa	2006a	An overview of reviews.
Tanner-Smith et al.	2016	No relevant outcomes.
Andrews et al.	2018	Not focusing on juvenile offenders.
Aos et al.	2001	Not focusing on juvenile offenders.
Cox et al.	1995	Not focusing on juvenile offenders.
DuBois et al.	2011	Not focusing on juvenile offenders.
Farrington & Welsh	2003	Not a meta-analysis or a systematic review.
Lieb	1994	Not enough extractable data.

Lipsey & Cullen	2007	An overview of reviews.
Lipsey et al.	2010	Not a meta-analysis or a systematic review.
Lipsey & Wilson	1993	An overview of reviews.
Lundahl et al.	2008	Not focusing on juvenile offenders.
Mitchell	2005	Not measuring an effect of an intervention or treatment program.
Wilson et al.	2000	Not focusing on juvenile offenders.
Hall	1995	Not focusing on juvenile offenders.
Hanson et al.	2002	Not focusing on juvenile offenders.
Losel & Schmucker	2005	Not focusing on juvenile offenders.
Lowenkamp et al.	2006	Not focusing on juvenile offenders.
Lowenkamp et al.	2005	Not enough extractable data.
Pearson & Lipton	1999b	Not focusing on juvenile offenders.
Pearson et al.	2002	Not focusing on juvenile offenders.
Tong & Farrington	2006	Not focusing on juvenile offenders.
Wilson et al.	2005	Not focusing on juvenile offenders.
Wilson et al.	2006	Not focusing on juvenile offenders.
Gallagher et al.	1999	Not focusing on juvenile offenders.
Julian & Kilmann	1979	Not a meta-analysis or a systematic review.
Strang et al.	2013	Not focusing on juvenile offenders.
Visher et al.	2006	Not focusing on juvenile offenders.
Dowden et al.	2003	Not a meta-analysis or a systematic review.
Watson	2016	Not enough extractable data.
Butts	1995	Not measuring an effect of an intervention or treatment program.
Altschuler	1998	Not a meta-analysis or a systematic review.
Stanton & Meyer	1998	Not measuring an effect of an intervention or treatment program.
Camp & Thyer	1993	Not enough extractable data.
Gordan et al.	1998	Not measuring an effect of an intervention or treatment program.
Brown et al.	2001	Not measuring an effect of an intervention or treatment program.
Wilson et al.	2016	Not enough extractable data.
Johnson	1985	Not a meta-analysis or a systematic review.
Lipton et al.	1998	Not focusing on juvenile offenders.
Lipton et al.	2002a	Not focusing on juvenile offenders.
Lipton et al.	2002b	Not focusing on juvenile offenders.
Heilbrun et el.	2005	Not measuring an effect of an intervention or treatment program.
Barman	2004	Not a meta-analysis or a systematic review.
Yarish	1995	Not a meta-analysis or a systematic review.
Redondo et al.	1999	Not focusing on juvenile offenders.
Latimer et al.	2001	Not focusing on juvenile offenders.
DuBois et al.	2002	No relevant outcomes.
Abrams et al.	2014	Not a meta-analysis or a systematic review.
Daykin et al.	2012	Not enough extractable data.
Sander et al.	2012	No relevant outcomes.
Shaffer	2006	Not focusing on juvenile offenders.
Shaffer	2011	Not focusing on juvenile offenders.
Dowden & Andrews	1999	Not focusing on juvenile offenders.
Latimer et al.	2006	Not focusing on juvenile offenders.
Aos et al.	2006	Not a meta-analysis or a systematic review.
Henggeler	2004	Not a meta-analysis or a systematic review.
Greenwood	2008	Not a meta-analysis or a systematic review.
Hanson et al.	2002	Not focusing on juvenile offenders.
Hanson et al.	2009	Not focusing on juvenile offenders.

Schmucker & Losel	2015	Not focusing on juvenile offenders.
Redondo et al.	2001	Not focusing on juvenile offenders.
Lipsey	2012	Not a meta-analysis or a systematic review.
Lipsey	1998	Not a meta-analysis or a systematic review.
Losel & Koferl	1989	Not focusing on juvenile offenders.
Losel	1996	An overview of reviews.
Roberts and Camasso	1991	Not enough extractable data to calculate an effect size
Izzo	1987	Not enough extractable data to calculate an effect size
Izzo and Ross	1990	Not enough extractable data to calculate an effect size
Lipsey	1999a	Not enough extractable data to calculate an effect size
Lipsey	1999b	Not enough extractable data to calculate an effect size
Whitehead and Lab	1989	Not enough extractable data to calculate an effect size
Bedard	2004	Not enough extractable data to calculate an effect size

SUPPLEMENTAL TABLE E Characteristics of included meta-analyses and systematic reviews

Review	# Of Studies (K)	Total # of juveniles (n)	Treatment (n)	Control (n)	Average	% Male	% White	Program Modality	Comparison Group
**Bouchard & Wong (2017)**	14	3104	2125	979	Not reported	73%	73%	Specialized diversion intervention-teen court	Formal processing and other diversion programs
**Dopp et al. (2017)**	28	5564	Not reported	Not reported	14.98	71.84%	58.29%	Family-based treatment	Treatment as usual
**Weaver & Campbell (2015)**	30	6620	3114	3506	16.5	63%	Not reported	Shock incarceration and boot camp	Treatment as usual
**Van der Stouwe et al. (2014)**	22	4066	1890	1835	14.73	75.95	Not reported	Multisystemic treatment	Treatment as usual
**Wilson & Hoge (2013)**	45	78640	33095	45545	14.72	95.38%	42.59%	Diversion programs	Formal processing
**James et al. (2013)**	22	5764	Not reported	Not reported	16.75	87.32%	30.64%	Varied: treatment modality, systemic treatment, individual treatment, group therapy,	No treatment and treatment as usual
**Lipsey (2009)**	361	Not reported	Not reported	Not reported	15.5	87%	34%	Varied: counseling, multiple services, skill building programs, restorative justice, surveillance, deterrence, discipline, multiple coordinated services	Treatment as usual
**Bouchard & Wong (2018)**	13	Not reported	Not reported	Not reported	Not reported	Not reported	23.08%	Varied: reentry/ aftercare services and intensive supervision probation	Standard probation
**Wilson et al. (2003)**	305	Not reported	Not reported	Not reported	Not reported	97%	54%	Mainstream delinquency intervention	Treatment as usual

**Woolfenden et al. (2002)**	7	749	Not reported	Not reported	Not reported	Mostly male	Not reported	Varied: family and parenting intervention, multisystemic therapy and multidimensional foster care	Treatment as usual
**Genoves et al. (2006)**	17	5833	2831	3002	16	Not reported	Not reported	Varied: cognitive-behavioral, cognitive, education, non-behavioral	Treatment as usual
**Latimer (2001)**	35	17141	10177	6964	Not reported	Not reported	Not reported	Family intervention	Treatment as usual
**Bradshaw et al. (2006)**	15	9172	Not reported	Not reported	Not reported	Not reported	Not reported	Victim offender mediation	Treatment as usual
**Schwalbe et al. (2012)**	28	19301	Not reported	Not reported	14.2	88%	91.50%	Varied: case management, individual treatment, family treatment, youth court, restorative justice, behavioral approaches, mentoring, crisis intervention	Treatment as usual
**Lipsey (1992a)**	397	Not reported	Not reported	Not reported	Not reported	Not reported	Not reported	Delinquency treatment	Treatment as usual
**Kettrey & Lipsey (2018)**	8	802	397	405	13.4 to 15.4	97.88%	Not reported	Psychosocial therapeutically oriented treatment	Treatment as usual
**Wong et al. (2016)**	21	18258	5209	13049	Not reported	88%	56%	Face-to-face Mediation	Treatment as usual
**Stein et al. (2015)**	31	8500	4250	4250	Not reported	79.20%	61.30%	Drug courts	Formal processing
**Koehler et al. (2012)**	21	7940	3883	4057	17.9	Not reported	Not reported	Varied: behavioral/cognitive–behavioral, intensive supervision/deterrence, and non-behavioral	Treatment as usual
**Stein et al. (2013)**	41	7559	Not reported	Not reported	Not reported	Not reported	60%	Drug court	Pre-treatment and treatment as usual

**Bradshaw & Roseborough (2005)**	5	2880	Not reported	Not reported	Not reported	Not reported	Not reported	Family group conferencing	Treatment as usual
**Walker et al. (2004)**	10	644	Not reported	Not reported	Not reported	100%	Not reported	Varied: multi systemic therapy, cognitive behavioral therapy, psychoeducational therapy, satiation therapy, vicarious sensitization, eclectic, residential	Treatment as usual
**Dowden & Andrews (2003)**	38	Not reported	Not reported	Not reported	Not reported	76%	Not reported	Family intervention	Treatment as usual
**Garrett (1985)**	111	13055	8076	4979	15.8	Not reported	Not reported	Varied	Treatment as usual
**Wilson et al. (2017)**	84	Not reported	Not reported	Not reported	Not reported	Not reported	Not reported	All restorative justice	Formal processing
**Garrido & Morales (2007)**	17,30	7509, 6658	3685	3824	16	Not reported	Not reported	Varied-individualized treatment	Treatment as usual
**Steele et al. (2016)**	18	8062	3612	4450	Not reported	Varied	Varied	Varied-education, remedial academic instruction, computer assisted instruction, personalized academic instruction, vocational, GED completion	Treatment as usual
**Dowden & Andrews (1999)**	134	Not reported	Not reported	Not reported	Not reported	84%	Not reported	Varied: Behavioral, Non-Behavioral	Control Group
**Nugent et al. (2004)**	15	9307	Not reported	Not reported	Not reported	Not reported	Not reported	Victim offender mediation	Treatment as usual

**Wilson & Lipsey (2000)**	28	3000	Not reported	Not reported	Not reported	Mostly male	Not reported	Wilderness challenge programs	Treatment as usual
**Tanner-Smith et al. (2016)**	32	8738	Not reported	Not reported	15.93	79%	67%	Drug court	Formal processing
**Petrosino et al. (2012)**	9	946	436	358	15-17	97%	52%	Scared straight	No treatment and treatment as usual
**Wilson et al. (2018a)**	19	11343	6274	5069	Not reported	Mostly male	Mostly white	Police initiated diversion	Treatment as usual
**Wilson et al. (2018b)**	6	Not reported	Not reported	Not reported	16.24	Not reported	49.10%	Trauma informed intervention (MTFC, TARGERT, CPT, Imagery Rehearsal Therapy, sanctuary model)	Treatment as usual
**Tolan et al. (2013)**	46	13335	6752	6583	Not reported	Not reported	cannot tell	Mentoring program	Treatment as usual
**Mayer et al. (1986)**	34	Not reported	Not reported	Not reported	14.58	81%	Not reported	Varied: behavioral treatment, token economy, positive reinforcement, modeling	Pre-treatment and treatment as usual
**Gensheimer et al. (1986)**	44	Not reported	Not reported	Not reported	14.6	73%	Not reported	Varied: behavioral, educational, individual, casework/probation	Pre-treatment and treatment as usual
**Gottschalk et al. (1987a)**	90	Not reported	Not reported	Not reported	14.6	76%	Not reported	Varied: behavioral, group, casework/probation, educational, individual.	Pre-treatment and treatment as usual
**Lipsey and Wilson (1998)**	200	Not reported	Not reported	Not reported	14 to 17	Mostly male	Not reported	Varied: interpersonal skills, individual counseling, behavioral program, multiple service	Control Group
**Gottschalk et al. (1987b)**	25	Not reported	Not reported	Not reported	15.7	78%	Not reported	Behavioral Approaches	Pre-treatment and treatment as usual
**Lipsey (1992b)**	443	Not reported	285 studies	131 studies	15	Mostly male	32.30%	Varied	Treatment as usual

**Ndrecka et al. (2009)**	34	7188	Not reported	Not reported	Not reported	Mostly male	12%	Varied: cognitive behavioral therapy, family functional therapy, mixed, multi-systematic therapy	Control Group
**Latimer et al. (2003)**	154	74518	30184	44334	15.23	Mostly male	Not reported	Varied: multi-focused, family-focused, individual-focused, institutional/residential, restorative justice, intensive supervision, group-focused, mixed/unknown, boot camps, wilderness programs.	Treatment as usual
**Winokur et al. (2006)**	11	813	406	407	14.9 to 16.9	Mostly male	Varied	Cognitive behavioral therapy	No treatment and treatment as usual
**Reitzel & Carbonell (2006)**	9	2986	1301	1685	14.6	87.21%	59%	Sex offender treatment: Varied- CBT, psychological, educational, psychotherapeutic, MST	Treatment as usual
**Livingstone et al. (2013)**	4, 16 citations	1447	Not reported	Not reported	Not reported	Not reported	Not reported	Restorative justice conferencing	Treatment as usual
**Petrosino et al. (2010)**	29	7779	3006	4773	14.73	74.20%	61.10%	Diversion or diversion with services	Formal processing
**Armelius & Andreassen (2007)**	12	4822	2439	2383	15-16	Mostly male	Not reported	Cognitive behavioral therapy	Control group and other intervention

SUPPLEMENTAL TABLE F Results from the fixed effects overall and moderator analyses

	Adjusted Effects[a]				Unadjusted Effects[b]			
	r$_\Phi$	Qb<br>95% CI	% Change	*k*	*r*$_\Phi$	Qb<br>95% CI	% Change	*k*
**Overall phi**	-0.105	(-0.107, -0.103)	-21.00%	56	-0.105	(-0.107, -0.103)	-21.00%	56
**Theoretical Moderators**								
Offender Type		2482.982***				669.857***		
Drug	-0.015	(-0.028, -0.003)	-3.08%	4	-0.025	(-0.037, -0.013)	-5.03%	4
General	-0.034	(-0.041, -0.028)	-6.88%	32	-0.106	(-0.108, -0.104)	-21.18%	32
Non-serious/non-violent	-0.067	(-0.076, -0.057)	-13.30%	8	-0.072	(-0.081, -0.063)	-14.37%	8
Serious/violent	-0.069	(-0.080, -0.057)	-13.70%	8	-0.086	(-0.095, -0.076)	-17.17%	8
Sexual	-0.166	(-0.178, -0.155)	-33.20%	4	-0.214	(-0.224, -0.204)	-42.76%	4
CJ System Setting		2297.999**				443.778***		
Institutionalized	-0.080	(-0.087, -0.074)	-16.08%	33	-0.119	(-0.122, -0.117)	-23.87%	33
Noninstitutionalized	-0.026	(-0.032, -0.020)	-5.18%	30	-0.067	(-0.071, -0.062)	-13.34%	30
CJ System Penetration		2444.257**				156.373***		
Correctional	-0.061	(-0.070, -0.053)	-12.20%	14	-0.062	(-0.067, -0.058)	-12.47%	14
Diversion	-0.036	(-0.075, 0.002)	-7.28%	19	-0.085	(-0.090, -0.080)	-17.02%	19
Reentry/Aftercare	-0.038	(-0.045, -0.031)	-7.60%	8	-0.025	(-0.033, -0.017)	-5.03%	8
Program Modality		3741.649***				2740.683***		
Cognitive behavioral treatment	-0.049	(-0.057, -0.040)	-9.70%	10	-0.049	(-0.057, -0.040)	-9.74%	10
Diversion treatment	-0.054	(-0.064, -0.043)	-10.70%	4	-0.067	(-0.075, -0.058)	-13.30%	4
Educational	-0.042	(-0.079, -0.004)	-8.32%	3	-0.041	(-0.079, -0.004)	-8.27%	3
Family-based treatment	-0.119	(-0.131, -0.107)	-23.80%	5	-0.147	(-0.150, -0.145)	-29.49%	5
Intensive supervision probation	0.004	(-0.055, 0.062)	0.74%	2	0.004	(-0.055, 0.062)	0.74%	2
Multisystemic treatment	-0.347	(-0.365, -0.328)	-69.30%	4	-0.358	(-0.371, -0.345)	-71.60%	4
Restorative justice	-0.127	(-0.140, -0.114)	-25.40%	6	-0.152	(-0.161, -0.144)	-30.49%	6
Shock incarceration	0.023	(0.009, 0.038)	4.68%	2	-0.005	(-0.015, 0.005)	-0.98%	2
Specialized courts	-0.030	(-0.044, -0.017)	-6.08%	6	-0.026	(-0.038, -0.014)	-5.24%	6
Wilderness therapy	-0.096	(-0.139, -0.053)	-19.24%	1	-0.090	(-0.131, -0.048)	-18.00%	1
AMSTAR Grade		1862.419***				1859.772***		
Low	-0.126	(-0.145, -0.140)	-25.18%	11	-0.055	(-0.060, -0.049)	-10.91%	11
Moderate	-0.055	(-0.059, -0.051)	-25.18%	21	-0.141	(-0.144, -0.139)	-28.28%	21
High	-0.055	(-0.060, -0.049)	-11.00%	24	-0.055	(-0.058, -0.051)	-10.98%	24

*$p < 0.05$, **$p < 0.01$, ***$p < 0.001$
[a]Adjusted effects = meta-regression results controlling for publication bias and methodological quality
[b]Unadjusted effects = traditional moderator analysis without methodological covariates

SUPPLEMENTAL TABLE G Fixed effects pairwise comparison matrix for type of offender moderator

	Reference Group				
**Comparison Group**	General	Sexual	Serious	Nonserious	Drug
General	--	0.000	0.000	0.000	0.003
Sexual	-0.132	--	0.000	0.000	0.000
Serious	-0.034	0.098	--	0.769	0.000
Nonserious	-0.032	0.100	0.002	--	0.000
Drug	0.019	0.151	0.053	0.051	--

Note. Numbers below the diagonal reflect the regression coefficient for each pairwise comparison; numbers above the diagonal reflect the *p*-value for each pairwise comparison.

SUPPLEMENTAL TABLE H Fixed effects pairwise comparison matrix for criminal justice system exposure moderator

	Reference Group		
**Comparison Group**	Diversion	Correctional	Reentry/Aftercare
Diversion	--	0.000	0.000
Correctional	-0.023	--	0.000
Reentry/Aftercare	0.066	0.089	--

Note. Numbers below the diagonal reflect the regression coefficient for each pairwise comparison; numbers above the diagonal reflect the *p*-value for each pairwise comparison.

SUPPLEMENTAL TABLE I Fixed effects pairwise comparison matrix for program modality moderator

	**Reference Group**									
**Comparison Group**	CBT	Diversion	Educational	Family	ISP	MST	RJ	Shock	SC	Wilderness
CBT	--	0.450	0.725	0.000	0.083	0.000	0.000	0.000	0.021	0.032
Diversion	-0.005	--	0.549	0.000	0.059	0.000	0.000	0.000	0.005	0.055
Educational	0.007	0.012	--	0.000	0.200	0.000	0.000	0.002	0.581	0.060
Family	-0.071	-0.066	-0.077	--	0.000	0.000	0.089	0.000	0.000	0.309
ISP	0.052	0.057	0.045	0.123	--	0.000	0.000	0.521	0.265	0.007
MST	-0.298	-0.293	-0.305	-0.228	-0.350	--	0.000	0.000	0.000	0.000
Restorative justice (RJ)	-0.079	-0.074	-0.085	-0.008	-0.131	0.220	--	0.000	0.000	0.173
Shock incarceration	0.072	0.077	0.065	0.142	0.020	0.370	0.150	--	0.000	0.000
Specialized courts (SC)	0.018	0.023	0.011	0.089	-0.034	0.316	0.100	-0.054	--	0.003
Wilderness	-0.048	-0.043	-0.055	0.022	-0.100	0.250	0.031	-0.120	-0.066	--

Note. Numbers below the diagonal reflect the regression coefficient for each pairwise comparison; numbers above the diagonal reflect the *p*-value for each pairwise comparison.

SUPPLEMENTAL TABLE J Fixed effects pairwise comparison matrix for AMSTAR moderator

	**Reference Group**		
**Comparison Group**	Low	Moderate	High
Low	--	0.000	0.000
Moderate	0.088	--	0.976
High	0.088	0.000	--

Note. Numbers below the diagonal reflect the regression coefficient for each pairwise comparison; numbers above the diagonal reflect the *p*-value for each pairwise comparison.

APPENDIX A

Abstract Screening Tool used to gather reports.

For all questions below, answer “yes”, “no”, or “maybe/unsure”.

Any question answered “no” is excluded.

Do not answer any further questions after the first “no”

1. Study Type:
    a. Is this a review of research? (e.g., meta-analysis, systematic review)
2. Language
    a. Is the abstract written in English?
3. Population
    a. Is this a study on juvenile/youth offenders?
    b. Are the participants between the ages of 10 and through age 25
4. Efficacy/Effectiveness Study
    a. Is the effect of a program being studied? (e.g., intervention, diversion, treatment, therapy, etc.)
5. Design
    a. Is a comparison group included in the study?
        i. Randomized control trial
        ii. Quasi-experimental design
        iii. comparison or Control group
        iv. treatment or Experimental group

Decision: Keep (all “yes” or “maybe/unsure” answers) or Drop (at least one “no” answer)

APPENDIX B

Full Text Screening Guide used to gather relevant reports.

For all questions below, answer “yes”, “no”, or “maybe/unsure”.
Any question answered “no” is excluded.
Do not answer any further questions after the first “no”

1. Study Type:
    a. Is this review a systematic review of research?

2. Language
    a. Is the full text written in English?

3. Population: Juvenile/youth offenders
    a. Does the review include participants anywhere between the ages of 10 and through age 25? (e.g., 12-18, 12-21, 10-18, 12-25, 12-22, etc.)

4. Efficacy/Effectiveness Study
    a. Is the effect of a program being studied? (e.g., intervention, diversion, treatment, therapy, reentry/aftercare, etc.)

5. Design
    a. Does the review include studies with a comparison group?
        i. Randomized control trial
        ii. Quasi-experimental design
        iii. comparison or Control group
        iv. treatment or Experimental group

6. Method Section of Review:
    a. Does the review include selection criteria?
    b. Does the review include at least one criminality outcome measure?
    c. Does the review include studies that are conducted in English?
    d. Does the review attempt to include unpublished literature in addition to published?
    e. If calculating effects, does the review report they type of effect size used?
    f. Does the review include a random effects model? (For meta-analysis only, not systematic review)

7. Results Section of Review:
    a. Does the review report results from the studies included?
    b. Does the review report results from the moderator analyses?
    c. Does the review include characteristics of the included studies?

8. Recidivism Measure:
    a. Is recidivism measured by any of the following?
        i. Alleged offenses (arrests, charges, referrals, court/police contacts) and convicted offenses (convictions, sustained petitions, adjudications, incarceration)

Decision: Keep (all “yes” or “maybe/unsure” answers) or Drop (at least one “no” answer)

APPENDIX C

Meta-review Coding Protocol

**Report Characteristics**
R1. What is the report ID number? (ID)
R2. What was the first author's last name? (NAME)
R3. What was the year of appearance of the report or publication? (YEAR)
R4. What type of report? (PUBTYPE)<br>book<br>book chapter<br>journal article<br>dissertation<br>thesis<br>private report<br>government report<br>conference paper<br>other (specify_____________________________)
R5. What type of review? (REVIEWTYPE)<br><br>meta-analysis<br>systematic Review<br>both<br>other (specify___________________________)<br><br>R6a. Year data collection started (DATASTART) (xxxx)<br>R6b. Year data collection ended (DATAEND) (xxxx)<br><br>R7. What type of literature does the review include? (LITERATURE)<br><br>published<br>unpublished<br>both<br>other (specify___________________________)<br><br>R7b. If the review includes unpublished literature, how many studies were unpublished? (UNPUBLISHED)<br><br>please specify the ratio of published to unpublished and percent.<br><br>R8. AMSTAR rating (AMSTAR)<br>high, moderate, low, critically low<br><br>R9. Is this review an update of a previous review? (either update or results reported elsewhere) (UPDATE)<br><br>please specify yes or no, and then describe what authors say

**Setting Characteristics**
S1. Geographic Location of the Studies included? (LOCATION)<br>united states

canada
uk
australia
eu
other (specify__________________)
varied (specify________________)

S2. Geographic location of the authors? (LOCATION2)
united states
canada
uk
australia
eu
other (specify__________________)
varied (specify________________)

S3. In what criminal justice setting was the study conducted? (CJSETTING)

community--diversion
community--post correctional facility
community--probation
community--aftercare
correctional facility
drug court
intensive-supervision probation
other (specify______________________________)
varied (specify________________)

S4. In what type of institution was the study conducted (INSTITUTION)
public
private
both
other (specify________________________)
varied (specify________________)

Participant (Study) Characteristics

P1. What is this sample ID number? (SAMPLE ID)

P2. Provide any "defining" characteristics of the sample. (DEFINING)

sex (specify ___________________________)
violent offenders (specify___________________________)
drug offenders (specify___________________________)
sexual offenders (specify _____________________________)
other (specify ___________________________________)
varied (specify________________)

P2b. What type of offenders are being studied? (OFFTYPE)

violent
serious
sexual
drug

general
other (specify_____________)
varied (specify_______________)

P3. Was the sample analyzed as subgroups? (SUBGROUP)

subgroup analysis of program type (specify _________)
subgroup analysis of offender type (specify _________)
subgroup analysis of SES (specify _________)
subgroup analysis of age (specify _________)
subgroup analysis of sex
subgroup analysis of timing of program initiation (specify _________)
subgroup analysis of publication type (specify___________)
subgroup analysis of research design (specify____________)
other subgroup analysis (specify _________)
varied (specify_______________)

P3b-c. If subgroup analysis:

subgroup name (GROUPNAME)
subgroup size (GROUPSIZE)

P4. What is the socio-economic status of juveniles included in the study? (SES)

low
middle
upper
multiple
other (specify___________________)
varied (specify_______________)

P4b. Specify as authors describe SES of sample. (SESDESCRIPT)

P5. What was the average age of juveniles included in the study? (AVERAGE)
(template: 12.5)

P6. What was the median age of juveniles? (MEDIAN)

P7. What was the youngest age of juveniles? (YOUNGEST)

P8. What was the oldest age of juveniles? (OLDEST)

P9. How many (number) studies are included in the review? (NUMINCLUDED)

P9b. What is the total number of youth/juveniles represented in the combined studies? (TOTAL)

P9c. What is the total number of youth/juveniles in the treatment? (TREATMENT)

P9d. What is the total number of youth/juveniles in the control? (CONTROL)

P10. What is the proportion of males in the review? (PROPMALES)

Please specify as the author's report.

P11. What racial or ethnic groups were represented in the sample? (ETHNICITY)

Specify the labels and percentages or proportions as reported.

P12. What is the proportion of Caucasians in the sample? (PROPWHITE)

Specify the labels and percentages or proportions as reported.

P13. What is the delinquency risk? (DELINQUENCY)

- low
- medium
- high

P14. How many juveniles in the study have a previous offense history? (HISTORY)

Specify the percentages, proportions, or means as reported.

Intervention Information:

I1. What type of intervention/program is being reviewed? (PROGRAMTYPE)

- diversion
  - restorative justice
  - victim-offender mediation
  - drug court
- correctional
  - education
  - cognitive-behavior therapy
  - religious
- reentry/aftercare
  - intensive supervision probation
- other (specify______________________________)
- varied (specify________________)

I1b. What is the intervention/program philosophy? (PHILOSOPHY)

- cbt for offenders
- boot camps
- drug courts
- drug treatment
- multisystemic treatment
- family intervention
- scared straight
- wilderness therapy
- sexual offender treatment
- educational program
- vocational program

mentoring
intensive supervision probation
employment
incarceration-based drug treatment
police diversion
other (specify____________________)
varied (specify________________)

I2. When was the intervention/program implemented? (TIMING)

after adjudication
before incarceration
during Incarceration
during probation
after incarceration
other (specify___________________________)
varied (specify________________)

I3. Was a researcher involved in the development of the intervention/program? (RESEARCHER)
yes
no

I4. What type of methodological design does the review include? (STUDYDESIGN)

experimental
quasi-experimental
both randomized and non-randomized
correlational
other (specify____________________)
varied (specify________________)

I5. What type of comparison group does the review include? (COMPARISON)

Specify as reported by the authors.
treatment as usual
no treatment control group
standard probation
formal/traditional processing
no treatment and treatment as usual
other diversion programs
control group
other (specify________________)
varied (specify________________)

I6. Does the review include a measurement of fidelity of implementation (yes/no)? (FIDELITY)

I6b. If so, what is the level of fidelity? (LEVEL)

low
medium

high
other (Specify_______________________)
varied (specify________________)

I7. Does the review include a measure of dosage (service amount--yes/no)? (DOSAGE)

I7b. If so, please specify as the authors reported (DOSAGELEVEL):
Duration:
Total hours:

I8. Is the program evidence-based by including principles of effective-interventions (yes/no)? (EVBASED)

I8b. Does the review include a measure of Risk-Need-Responsivity? (RNR)

I9. Which definition of a program is used? (PROGRAMDEF)

specific operating procedure of a particular program (sop)
brand name protocol program
generic intervention program
other (specify______________________)
varied (specify________________)

I9b. Please specify the type of program being used as provided by the authors. (EVBASEDTYPE)

For SOP:
please list name of specific program as described by author.
For brand name:
functional family therapy (FFT)
multisystemic therapy (MST)
multidimensional dimensional treatment foster care
aggression replacement training (ART)
other (specify_______________________)
varied (specify________________)

For generic intervention type:
family therapy
mentoring
cognitive behavior therapy
drug treatment
sex offender treatment
other (specify____________________)
varied (specify________________)

Outcome Information

O1. What is the Outcome ID Number (OUTCOMEID)

O2. What type of outcome measure is being reported (OUTCOMETYPE)

recidivism
educational attainment
job attainment
delinquency
other (specify___________________________________)
varied (specify________________)

O3. How is the outcome being measured operationally? (OUTCOMEMEASURE)
Specify as reported by the authors

O4. What is the follow up period for measuring the outcome? (FOLLOWUP)
Specify as reported by the authors.

O5. Source of outcome measure: (OVSOURCE) _______
self-report: paper & pencil or computer
self-report: personal interview
self-report: telephone interview
self-report: other
self-report: cannot tell
other report: parent
other report: peers
other report: teacher(s)
other report: therapist/service provider
other report: other
other report: cannot tell
records: school
records: police
records: probation
records: court
records: custodial institution
records: regional crime statistics
records: other
records: cannot tell
any other
cannot tell
other (specify___________________________________)
varied (specify________________)

EFFECT SIZE

E1. Effect Size Index (as labeled by authors) (ESINDEX)

cohens d
glass delta
hedges g
correlation coefficient
phi coefficient
odds ratio
risk ratio
besd
other (specify______________________________)

varied (specify________________)

E1b. Page Found (ESINDEXPG)

E2. Effect Size (as reported by the authors in meta-analysis) (EFFECT SIZE)

E2b. Confidence Interval (sometimes a Credible Interval) (95CI)
Please specify the range of the interval as reported (e.g. 0.8-1.2)

E2c. Page Found that confirms what it tells you meaningfully (ESSIZEPG)

E3. What type of effects model does the meta-analysis use? (EFFECTMODEL)

fixed effects
random effects
both
other (specify__________________________)
varied (specify________________)

E4. Does the review include a measure of heterogeneity (yes/no)? (HGENEITY)

E4b. If so, which measure do they use to calculate heterogeneity? (HGMEASURE)

q-statistic
i-squared
both
other (specify__________________________)
varied (specify________________)

E4c-d.

q-statistic size (QSTATSITIC)
i-squared size (ISQUARED)

Coder and Coding Characteristics

C1. What are your initials? (INITIALS)

C2. In minutes, approximately how long did it take you to code this study? (MINUTES)

C3. Provide any notes about the reports or concerns regarding your coding of it. (NOTES)
** Make sure to include all notes in a single cell and “drag them down” to all rows
