# Supplementary material for: The 40-year debate: a meta-review on what works for juvenile offenders
Source: J Exp Criminol. 2021 Jun 12;19(1):1–30. doi: 10.1007/s11292-021-09472-z (PMC8196268; doi:10.1007/s11292-021-09472-z)
Supplement: Supplementary file 3 — (PDF 107 kb) [file 11292_2021_9472_MOESM3_ESM.pdf]

**SUPPLEMENTAL FILE C**

Complete list of references included in the statistical analysis.


Armelius, B-A & Andreassen, T.H. (2007). Cognitive-behavioral treatment for antisocial behavior for youth in residential treatment. *Cochrane Database of Systematic Reviews, 2007*(4), 1-35.

Bouchard, J., & Wong, J. S. (2017). A Jury of Their Peers: A meta-analysis of the effects of teen court on criminal recidivism. *Journal of Youth and Adolescence*, *46*(7), 1472-1487.

Bouchard, J. & Wong, J. S. (2018). Examining the effects of intensive supervision and aftercare programs for at-risk youth: A systematic review and meta-analysis. *International Journal of Offender Therapy and Comparative Criminology, 62*(6), 1509-1534.

Bradshaw, W., & Roseborough, D. (2005). An empirical review of family group conferencing in juvenile offenses. *Juvenile and Family Court Journal,* 56(4), 21-48.

Bradshaw, W., Roseborough, D., & Umbreit, M. S. (2006). The effect of victim offender mediation on juvenile offender recidivism: A meta-analysis. *Conflict Resolution Quarterly, 24*(1), 87-98.

Dopp, A. R., Borduin, C. M., Kuppens, S., & White II, M. H. (2017). Family-based treatments for serious juvenile offenders: A multilevel meta-analysis. *Journal of Consulting and Clinical Psychology*, *85*(4), 335-354.

Dowden, C., & Andrews, D. A. (1999b). What works in young offender treatment: A meta-analysis. *Forum on Corrections Research*, *11*(2), 21-24.

Dowden, C., & Andrews, D. A. (2003). Does family intervention work for delinquents? Results of a meta-analysis. *Canadian Journal of Criminology and Criminal Justice, 45*(3), 327-342.

Garrett, C. J. (1985). Effects of residential treatment on adjudicated delinquents: A meta-analysis. *Journal of Research in Crime and Delinquency, 22*(4), 287-308.

Garrido, V., & Morales, L. A. (2007). Serious (violent or chronic) juvenile offenders: A systematic review of treatment effectiveness in secure corrections. *Campbell Systematic Reviews, 2007*(7), 1-46.

Genovés, V. G., Morales, L. A., & Sánchez-Meca, J. (2006). What works for serious juvenile offenders? A systematic review. *Psicothema, 18*(3), 611-619.

Gensheimer, L. K., Mayer, J. P., Gottschalk, R., Davidson, I. I., & William, S. (1986). Diverting youth from the juvenile justice system: A meta-analysis of intervention efficacy. In S. J. Apter & A. P. Goldstein (Eds.), *Pergamon general psychology series, Vol. 135. Youth violence: Programs and prospects* (pp. 39-57). Elmsford, NY, US: Pergamon Press.

Gottschalk, R., Davidson, W.S. I., Gensheimer, L. K., & Mayer, J. P. (1987a). Community-based interventions. In H. C. Quay (Ed.), *Wiley series on personality processes. Handbook of juvenile delinquency* (pp. 266-289). Oxford, England: John Wiley & Sons.

Gottschalk, R., Davidson, W.S.I., Mayer, J., & Gensheimer, L. K. (1987b). Behavioral approaches with juvenile offenders: A meta-analysis of long-term treatment efficacy. In E. K. Braukmann, *Behavioral approaches to crime and delinquency: A handbook of application, research, and concepts* (pp. 399-422). New York, NY: Plenum Press.

James, C., Stams, G.J., Asscher, J.J., Katrien De Roo, A., & Van der Laan, P.H. (2013). Aftercare programs for reducing recidivism among juvenile and young adult offenders: A meta-analytic review. *Clinical Psychology Review, 33*(2), 263-274.

Kettrey, H. H., & Lipsey, M. W. (2018). The effects of specialized treatment on the recidivism of juvenile sex offenders: A systematic review and meta-analysis. *Journal of Experimental Criminology, 14*(3), 361-387.

Koehler, J. A., Losel, F., Akoensi, T. D., & Humphreys, D. K. (2012). A systematic review and meta-analysis on the effects of young offender treatment programs in Europe. *Journal of Experimental Criminology, 9*(1), 19-43.

Latimer, J. (2001). A meta-analytic examination of youth delinquency, family treatment, and recidivism. *Canadian Journal of Criminology, 43*, 237-253.

Latimer, J. & Dowden, C. (2003). Treating youth in conflict with the law: A new meta-analysis. Canada: Youth Justice Research Series/Department of Justice Canada.

Lipsey, M. W. (1992a). The effect of treatment on juvenile delinquents: Results from meta-analysis. In F. Lösel, D. Bender, & T. Bliesener (Eds.), *Psychology and law: International perspectives* (pp. 131-143). Berlin: Walter de Gruyter.

Lipsey M. W. (1992b). Juvenile delinquency treatment: A meta-analytic inquiry into the variability of effects. In T. D. Cook, H. Cooper, D. S. Cordray, H. Hartmann. L. V. Hedges, R. J. Light, T. A. Louis, F. Mosteller (Eds.), *Meta-Analysis for Explanation* (pp. 83-127) New York, NY: Russell Sage Foundation.

Lipsey, M. W. (1995). What do we learn from 400 research studies on the effectiveness of treatment with juvenile delinquents? In J. McGuire (Ed.), *Wiley series in offender rehabilitation. What works: Reducing reoffending: Guidelines from research and practice* (p. 63–78). John Wiley & Sons.

Lipsey, M.W. (2009). The primary factors that characterize effective interventions with juvenile offenders: A meta-analytic overview. *Victims and Offenders, 4*(2), 124–147.

Lipsey, M. W., & Wilson, D. B. (1998). Effective intervention for serious juvenile offenders: A synthesis of research. In R. Loeber & D. P. Farrington (Eds.), *Serious & violent juvenile offenders: Risk factors and successful interventions* (pp. 313-345). Thousand Oaks, CA, US: Sage Publications, Inc.

Lipsey, M.W., Wilson, D.B., & Cothern L. (2000). Effective intervention for serious juvenile offenders. *Office of Juvenile Justice and Delinquency Prevention,* 1-8.

Livingstone, N., Macdonald, G., & Carr, N. (2013). Restorative justice conferencing for reducing recidivism in young offenders (aged 7 to 21). *Cochrane Database of Systematic Reviews, 2013*(2), 1-89.

Mayer, J. P., Gensheimer, L. K., Davidson, I. I., William, S., & Gottschalk, R. (1986). Social learning treatment within juvenile justice: A meta-analysis of impact in the natural environment. In S. J. Apter & A. P. Goldstein (Eds.), *Pergamon general psychology series, Vol. 135. Youth violence: Programs and prospects* (pp. 24-38). Elmsford, NY, US: Pergamon Press.

Ndrecka, M. M., Betchel, K. M., Lowenkamp, C. T., & Latessa, E. J. (2009). Effectiveness of Juvenile Cognitive Behavioral and Family-Oriented Interventions- A Meta-Analysis. In *Cognitive Behavioral-Interventions for At-Risk Youth* (pp. 14-24). Civic Research Institute, Inc.

Nugent, W.R., Williams, M., & Umbreit, M.S., (2003). Participation in victim-offender mediation and the prevalence and severity of subsequent delinquent behavior: A meta-analysis. *Utah Law Review, 137*(1), 137-166.

Nugent, W. R., Williams, M., & Umbreit, M.S. (2004). Participation in victim-offender mediation and the prevalence of subsequent delinquent behavior: A meta-analysis. *Research on Social Work Practice. 4*(6), 408-416.

Petrosino, A., Turpin-Petrosino, C., & Buehler, J. (2003). Scared straight and other juvenile awareness programs for preventing juvenile delinquency: A systematic review of the randomized experimental evidence. *The ANNALS of the American Academy of Political and Social Science, 589*(1), 41–62.

Petrosino, A., Turpin-Petrosino, C., & Finckenauer, J.O. (2000). Well-meaning programs can have harmful effects! Lessons from experiments of programs such as scared straight. *Crime & Delinquency, 46*(3), 354-379.

Petrosino A., Turpin-Petrosino C., & Guckenburg, S. (2010). Formal system processing of juveniles: Effects on delinquency. *Campbell Systematic Reviews, 2010*(1), 1-88.

Petrosino, A., Turpin-Petrosino, C., Hollis-Peel, M. E., & Lavenberg, J. G., (2012). Scared straight and other juvenile awareness programs for preventing juvenile delinquency: a systematic review. *Campbell Systematic Reviews,* 2013(5), 1-55.

Reitzel, L. R., & Carbonell, J. L. (2006). The effectiveness of sexual offender treatment for juveniles as measured by recidivism: A meta-analysis. *Sexual Abuse, 18*(4), 401-421.

Schwalbe, C. S., Gearing, R. E., MacKenzie, M. J., Brewer, K. B., & Ibrahim, R. (2012). A meta-analysis of experimental studies of diversion programs for juvenile offenders. *Clinical Psychology Review, 32*(1), 26–33.

Steele, J. L., Bozick, R., & Davis, L. M. (2016). Education for incarcerated juveniles: A meta-analysis. *Journal of Education for Students Placed at Risk (JESPAR), 21*(2), 65-89.

Stein, D. M., Deberard, S., & Homan, K. (2013). Predicting success and failure in juvenile drug treatment court: A meta-analytic review. *Journal of Substance Abuse Treatment*, *44*, 159-168.

Stein, D. M., Homan K. J., & DeBerard, S. (2015) The Effectiveness of Juvenile Treatment Drug Courts: A Meta-Analytic Review of Literature, *Journal of Child & Adolescent Substance Abuse, 24*(2), 80-93.

Tanner-Smith, E. E., Lipsey, M. W., & Wilson, D. B. (2016a). Juvenile drug court effects on recidivism and drug use: A systematic review and meta-analysis. *Journal of Experimental Criminology*, *12*(4), 477-513.

Tanner-Smith, E. E., Lipsey, M. W., & Wilson, D. B. (2016b). Meta-Analysis of Research on the Effectiveness of Juvenile Drug Courts. Nashville, TN: Peabody Research Institute, Vanderbilt University.

Tolan, P., Henry, D., Schoeny, M., Bass, A., Lovegrove, P., & Nichols, E. (2013). Mentoring interventions to affect juvenile delinquency and associated problems: A systematic review. *Campbell Systematic Reviews*, *2013*(2),1-148.

Van der Stouwe, T., Asscher, J. J., Stams, G. J., Deković, M., & Laan, P. H. (2014). The effectiveness of Multisystemic Therapy (MST): A meta-analysis. *Clinical Psychology Review*, *34*(6), 468-481.

Walker, D. F., Mcgovern, S. K., Poey, E. L., & Otis, K. E. (2004). Treatment effectiveness for male adolescent sexual offenders: A meta-analysis and review. *Journal of Child Sexual Abuse*, *13*(3-4), 281-293.

Weaver, R.D. & Campbell, D. (2014). Fresh start: A meta-analysis of aftercare programs for juvenile offenders. *Research on Social Work Practice*, *25*(2), 201-212.

Wilson, D. B., Brennan, I., & Olaghere, A. (2018a). Police-initiated diversion for youth to prevent future delinquent behavior: A systematic review. *Campbell Systematic Reviewa. 2018*(5), 1-85.

Wilson, H.A., & Hoge, R. D. (2013). The effect of youth diversion programs on recidivism. *Criminal Justice and Behavior, 40*(5), 497-518.

Wilson, D. B., Olaghere, A., & Kimbrell, C. S., (2017). Effectiveness of restorative justice principles in juvenile justice: A meta-analysis. Retrieved from http://www.ncjrs.gov/App/publications/abstract.aspx?ID=273052

Wilson, D.B., Olaghere, A., & Kimbrell, C. S. (2018b). Trauma-focused interventions for justice-involved and at-risk youth: A meta-analysis. *U.S. Department of Justice Office of Justice Programs.*

Wilson, S. J., & Lipsey, M. W. (2000). Wilderness challenge programs of delinquent youth: A meta-analysis of outcome evaluations. *Evaluation and Program Planning. 23*(1). 1-12.

Wilson, S.J., Lipsey, M. W., & Soydan, H. (2003). Are mainstream programs for juvenile delinquency less effective with minority youth than majority youth? A meta-analysis of outcomes research. *Research on Social Work Practice*, *13*(1), 3-26.

Winokur, M., Rozen, D., Batchelder, K., & Valentine, D. (2006). *Juvenile Sexual Offender Treatment: A Systematic Review of Evidence-Based Research*. (Final Report) Fort Collins, CO: Social Work Research Center.

Wong, J. S., Bouchard, J., Gravel, J., Bouchard, M., & Morselli, C. (2016). Can at-risk youth be diverted from crime? A meta-analysis of restorative diversion programs. *Criminal Justice and Behavior. 43*(10), 1310-1329.

Woolfenden, S. R., Williams, K., & Peat, J. K. (2002). Family and parenting interventions for conduct disorder and delinquency: A meta-analysis of randomized controlled trials. *Archives of Disease in Childhood, 86*, 251-256.
